# Supplementary figures and images for: Synthesis, antibacterial activity, in silico ADMET prediction, docking, and molecular dynamics studies of substituted phenyl and furan ring containing thiazole Schiff base derivatives
Source: PLoS One. 2025 Mar 10;20(3):e0318999. doi: 10.1371/journal.pone.0318999 (PMC11892886; doi:10.1371/journal.pone.0318999)

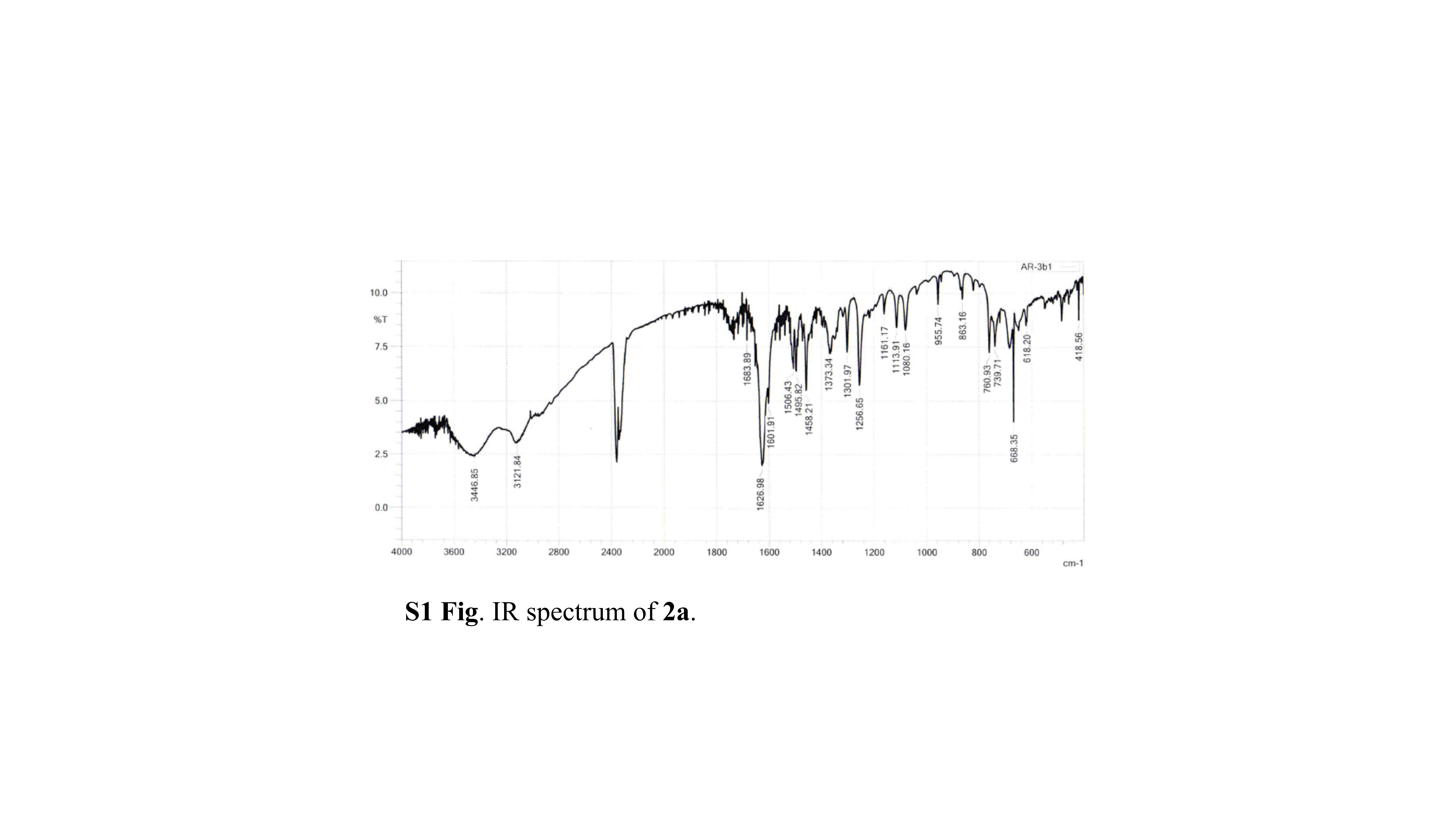

Supplement: S1 Fig — (TIF) [file pone.0318999.s001.tif]

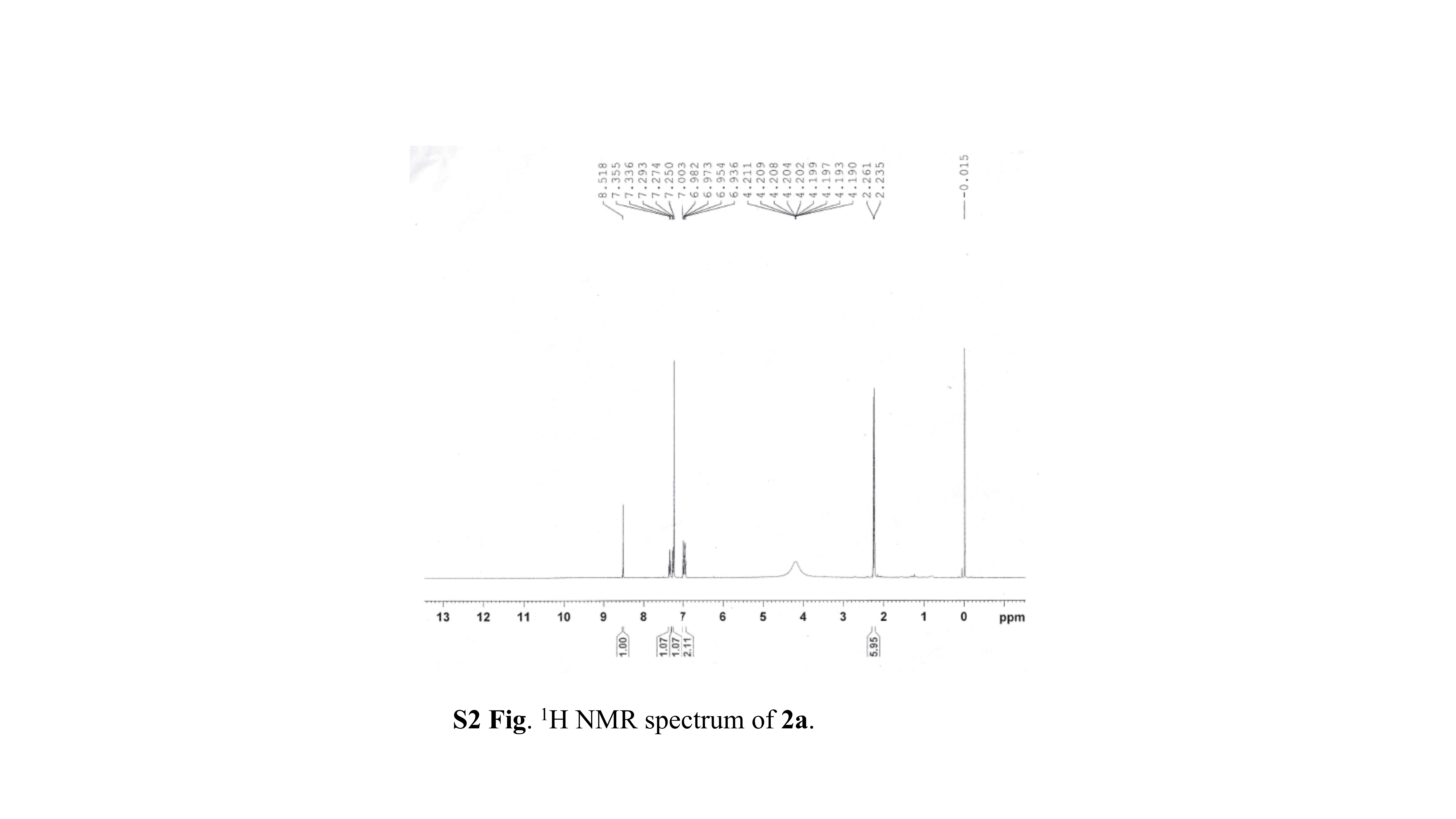

Supplement: S2 Fig — 1H NMR spectrum of 2a. (TIF) [file pone.0318999.s002.tif]

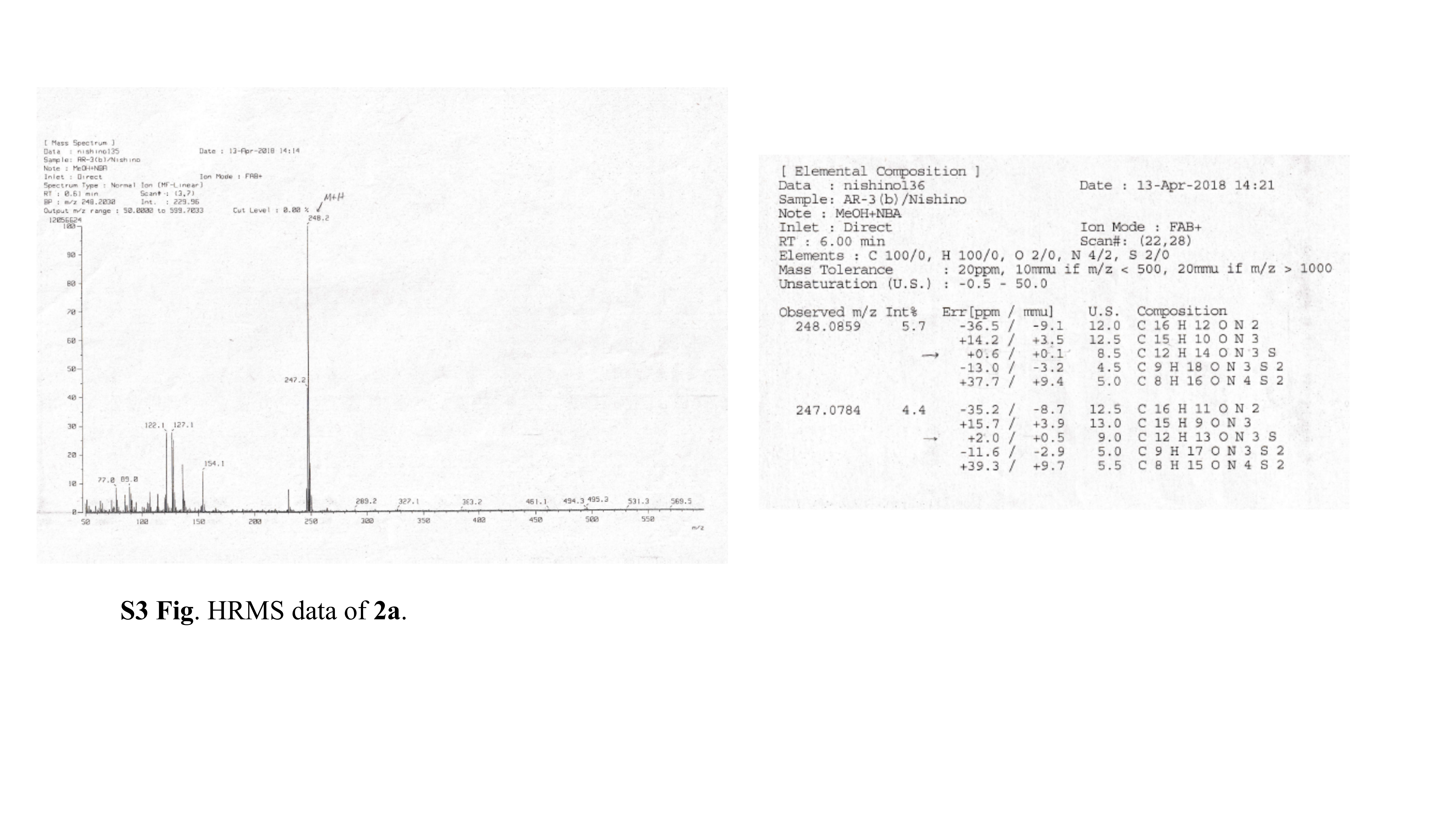

Supplement: S3 Fig — (TIF) [file pone.0318999.s003.tif]

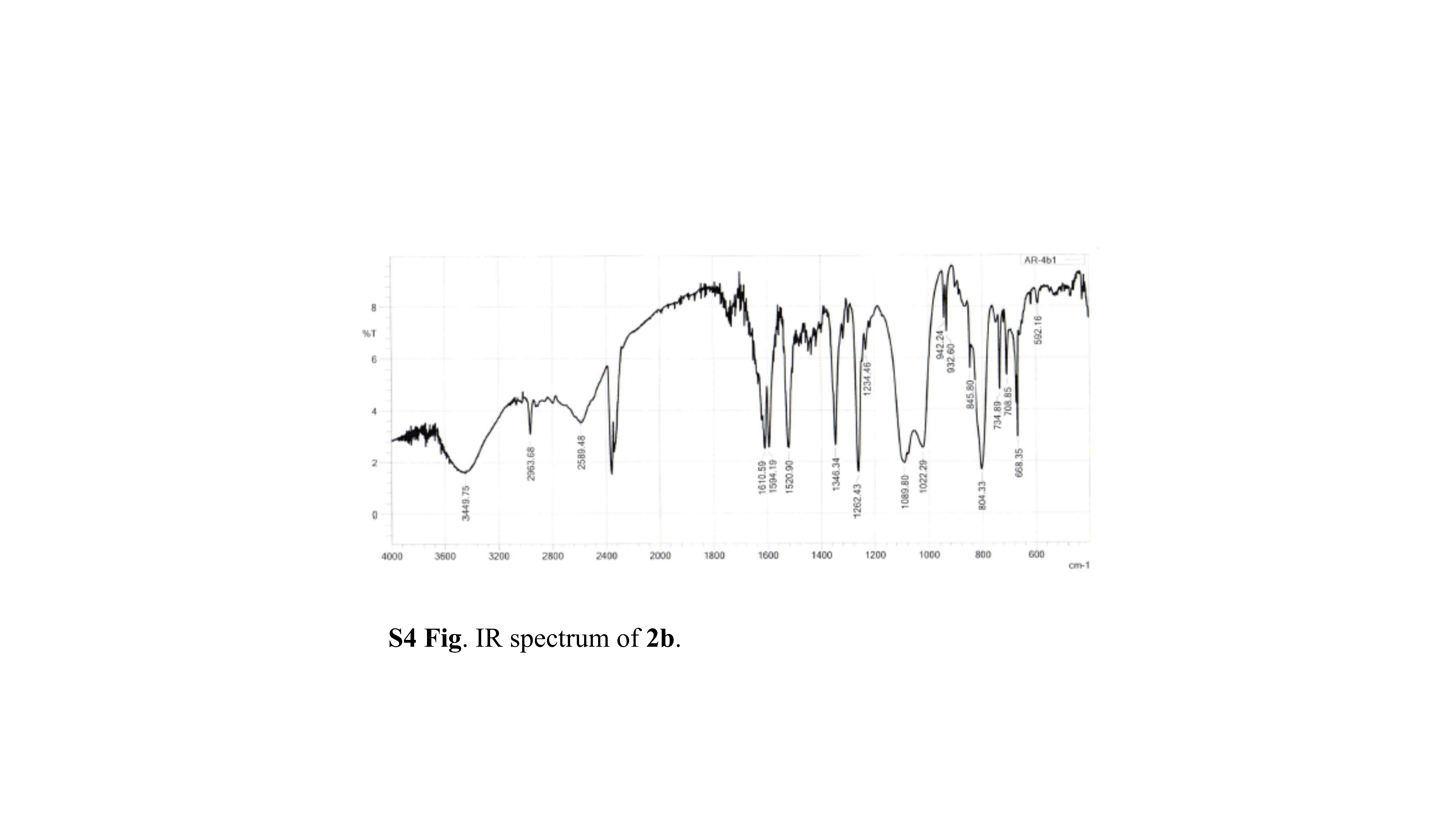

Supplement: S4 Fig — (TIF) [file pone.0318999.s004.tif]

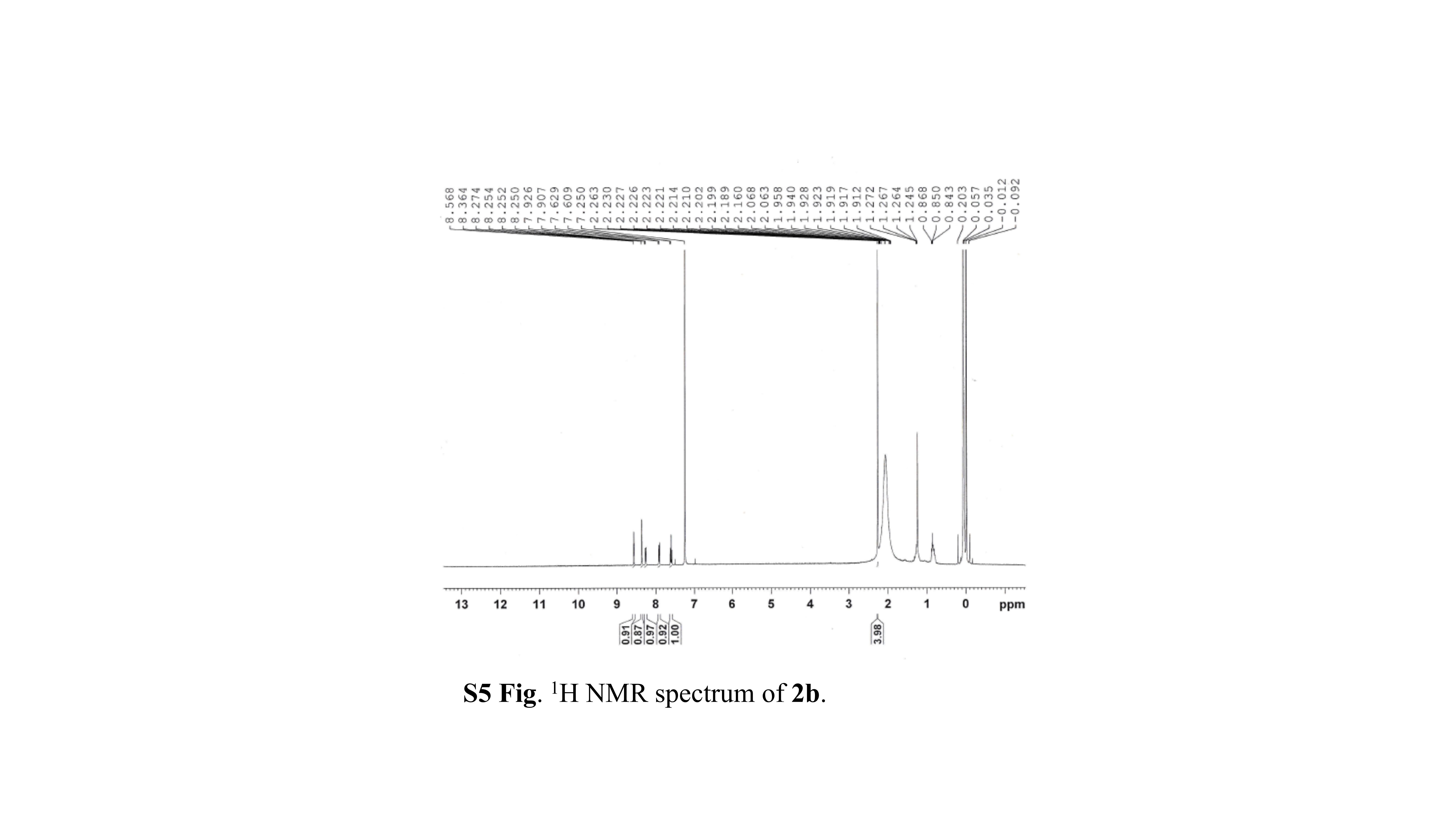

Supplement: S5 Fig — 1H NMR spectrum of 2b. (TIF) [file pone.0318999.s005.tif]

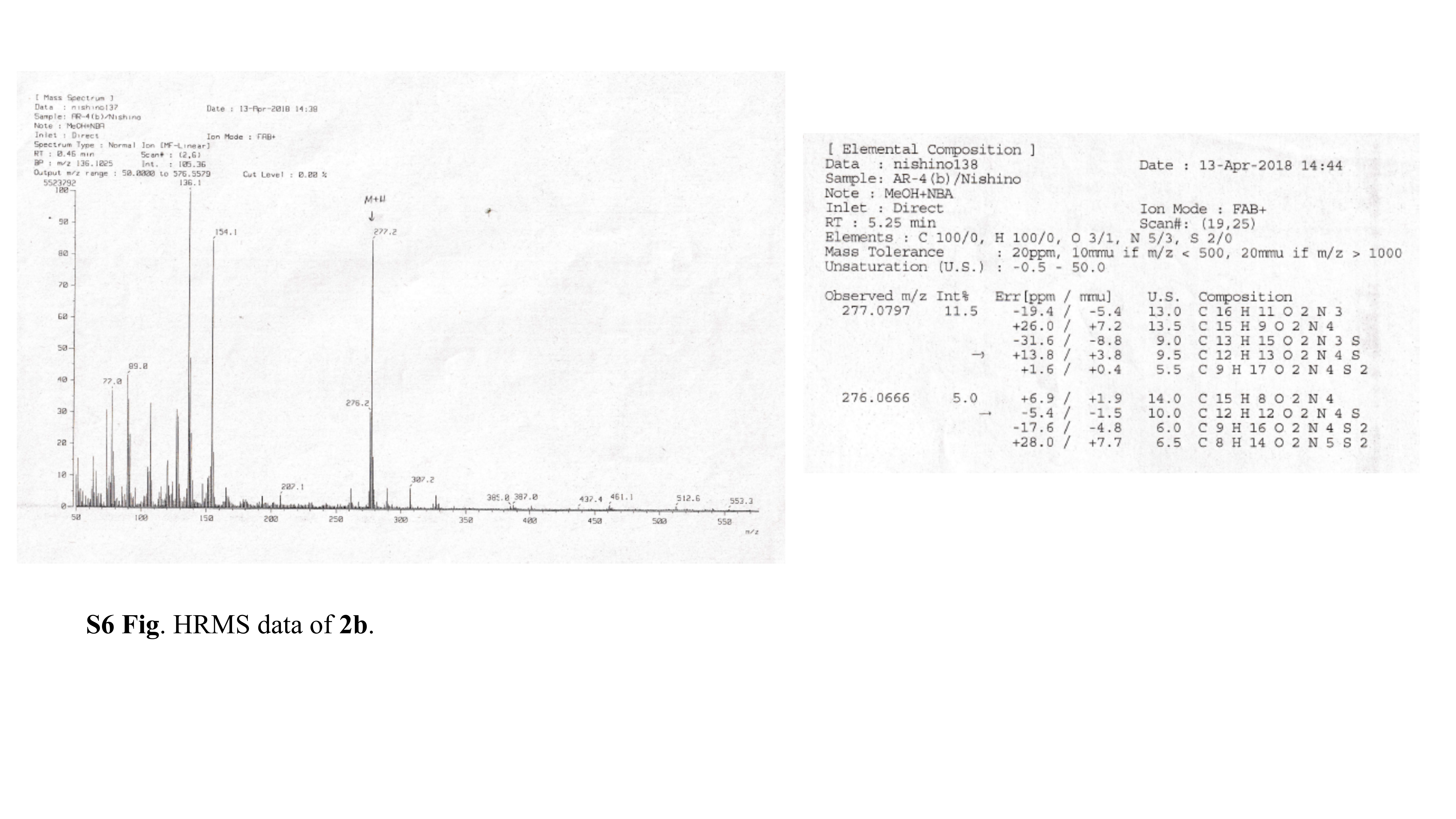

Supplement: S6 Fig — (TIF) [file pone.0318999.s006.tif]

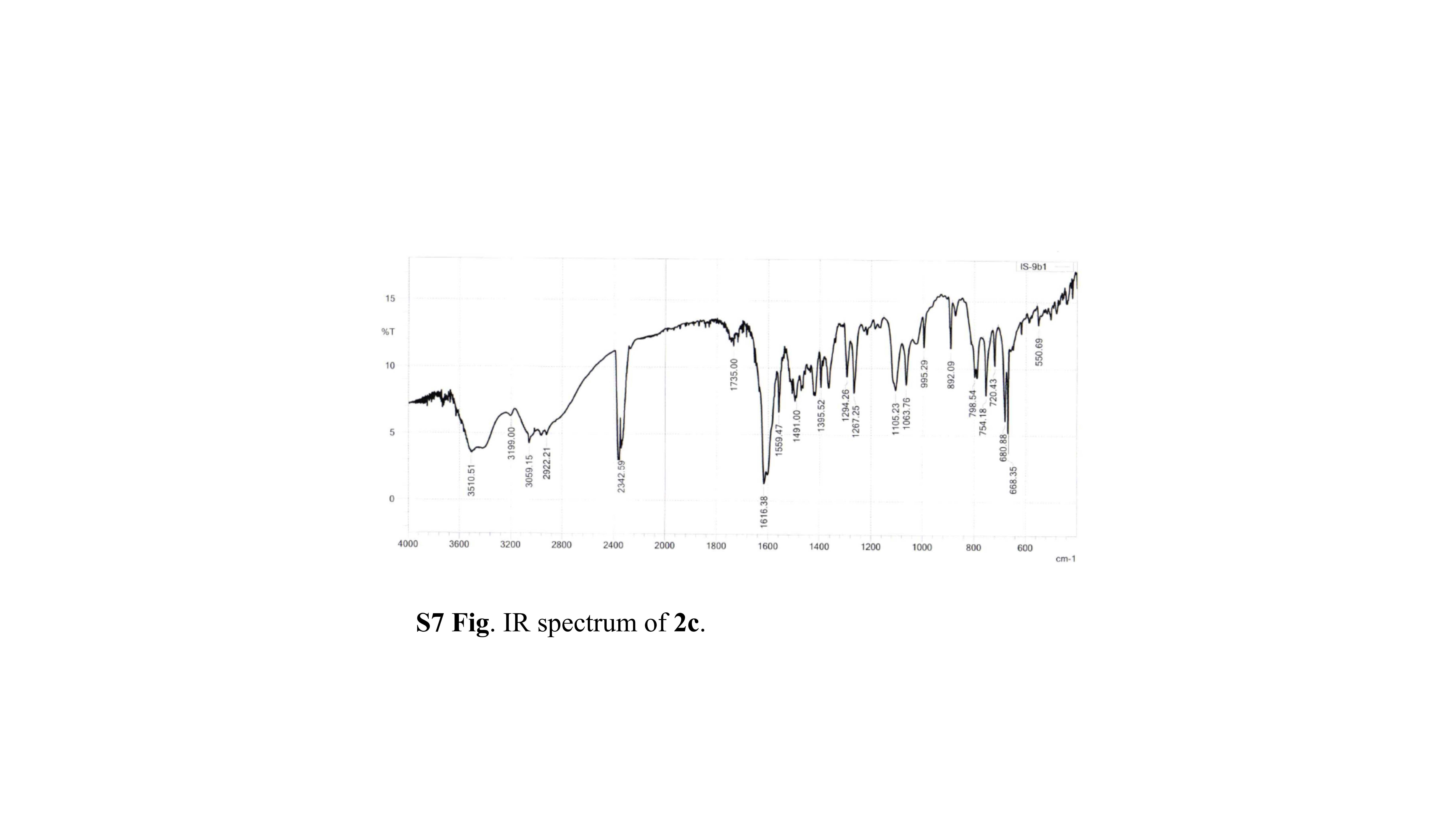

Supplement: S7 Fig — (TIF) [file pone.0318999.s007.tif]

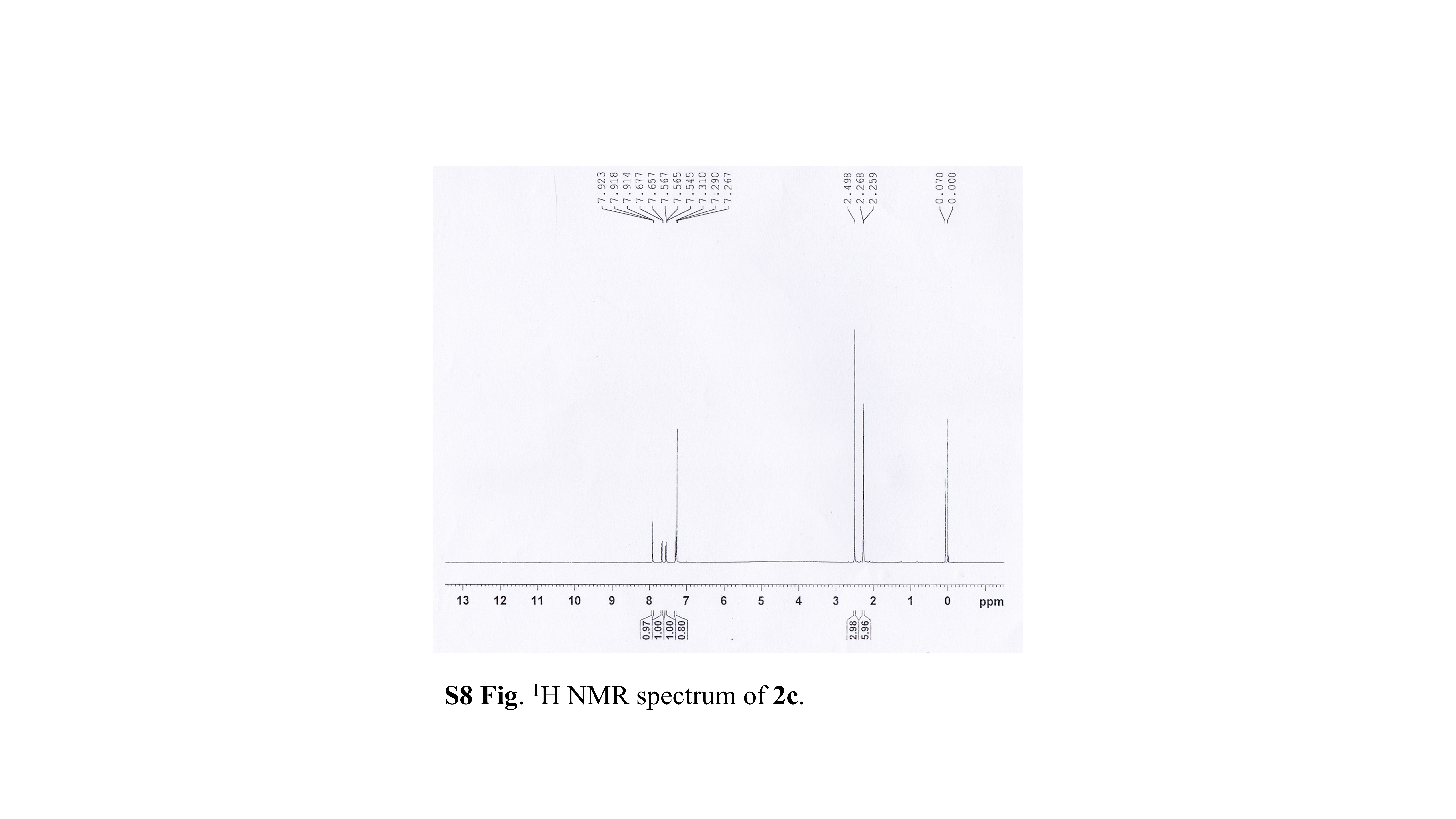

Supplement: S8 Fig — 1H NMR spectrum of 2c. (TIF) [file pone.0318999.s008.tif]

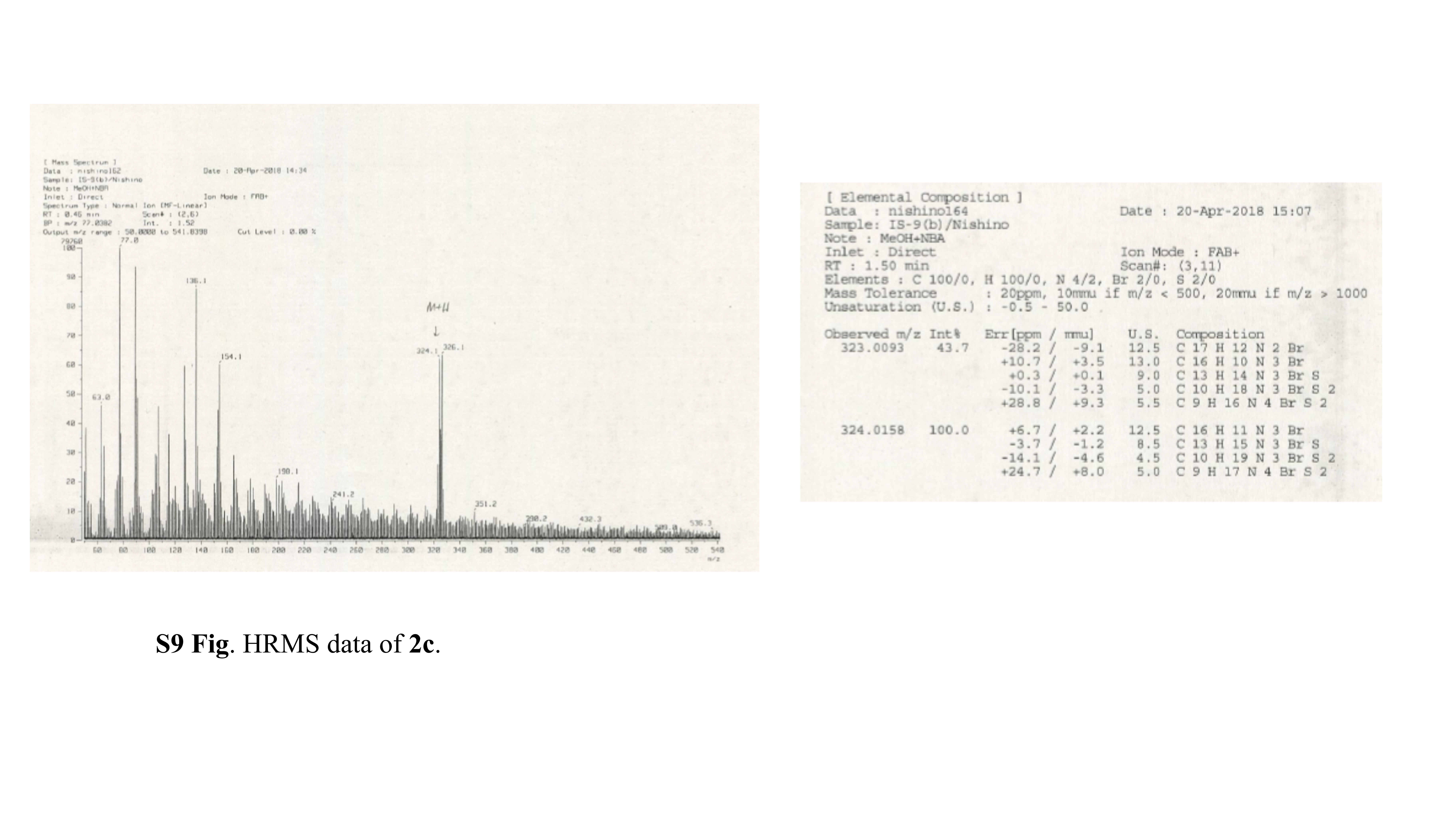

Supplement: S9 Fig — (TIF) [file pone.0318999.s009.tif]

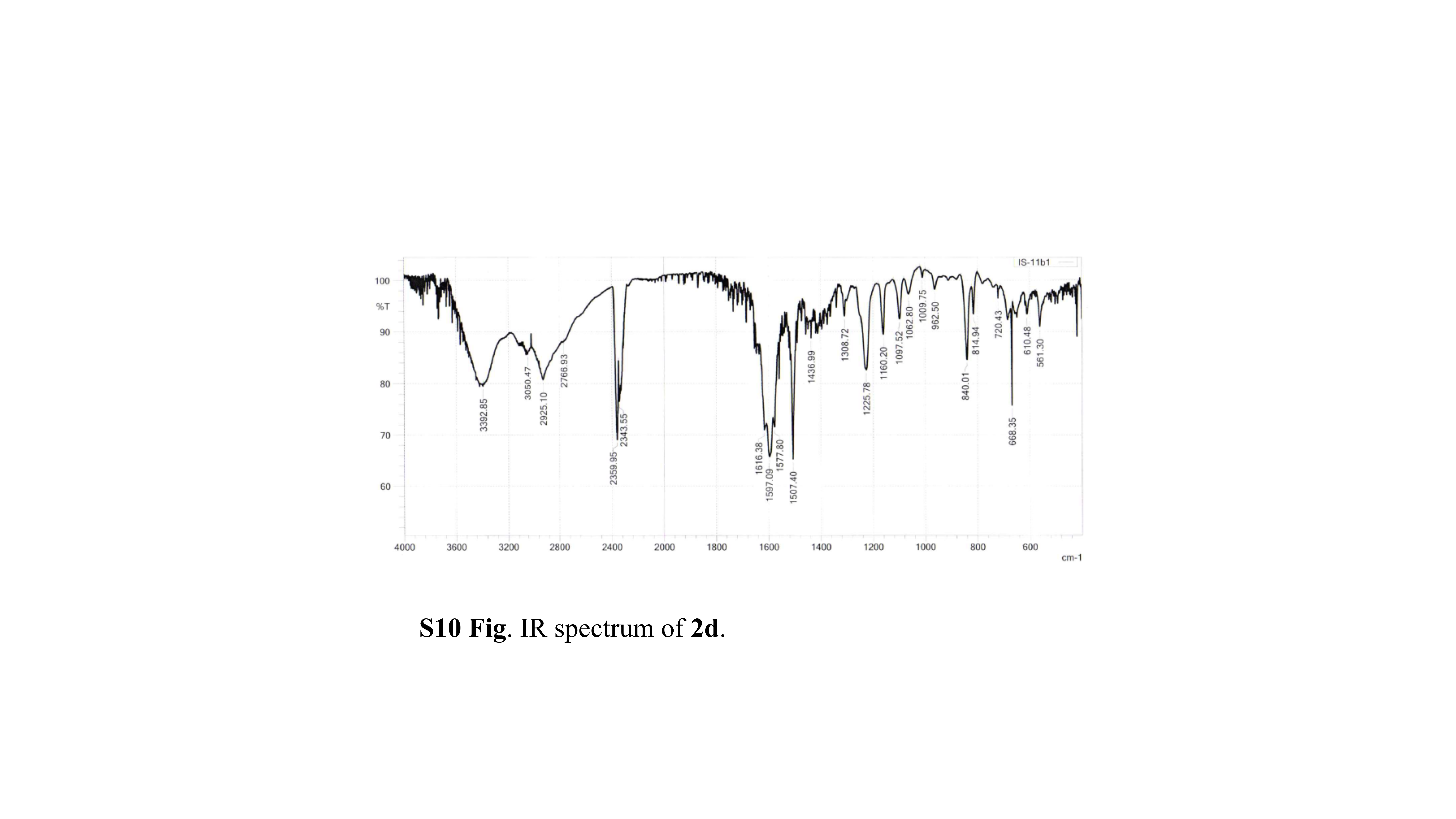

Supplement: S10 Fig — (TIF) [file pone.0318999.s010.tif]

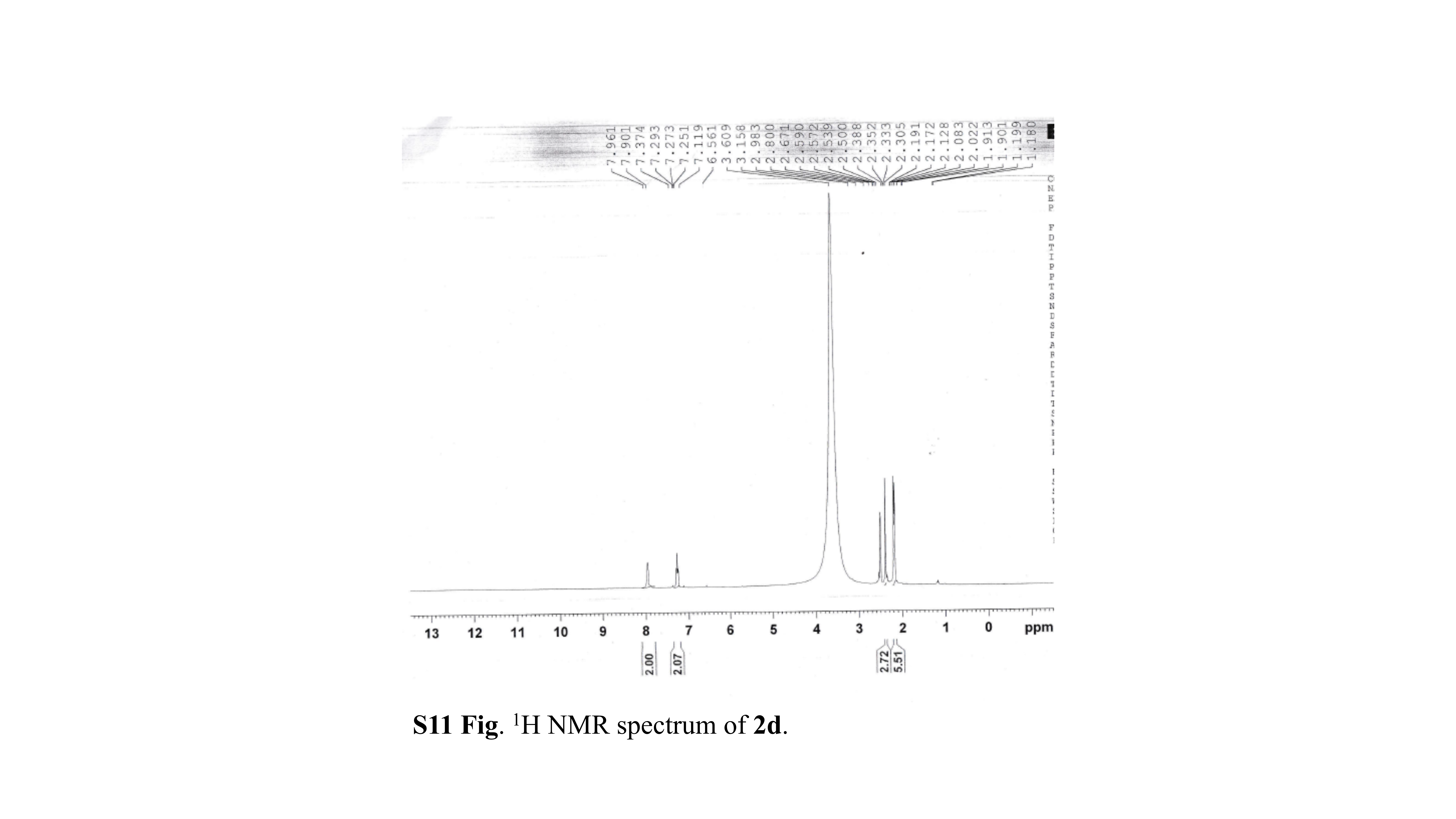

Supplement: S11 Fig — 1H NMR spectrum of 2d. (TIF) [file pone.0318999.s011.tif]

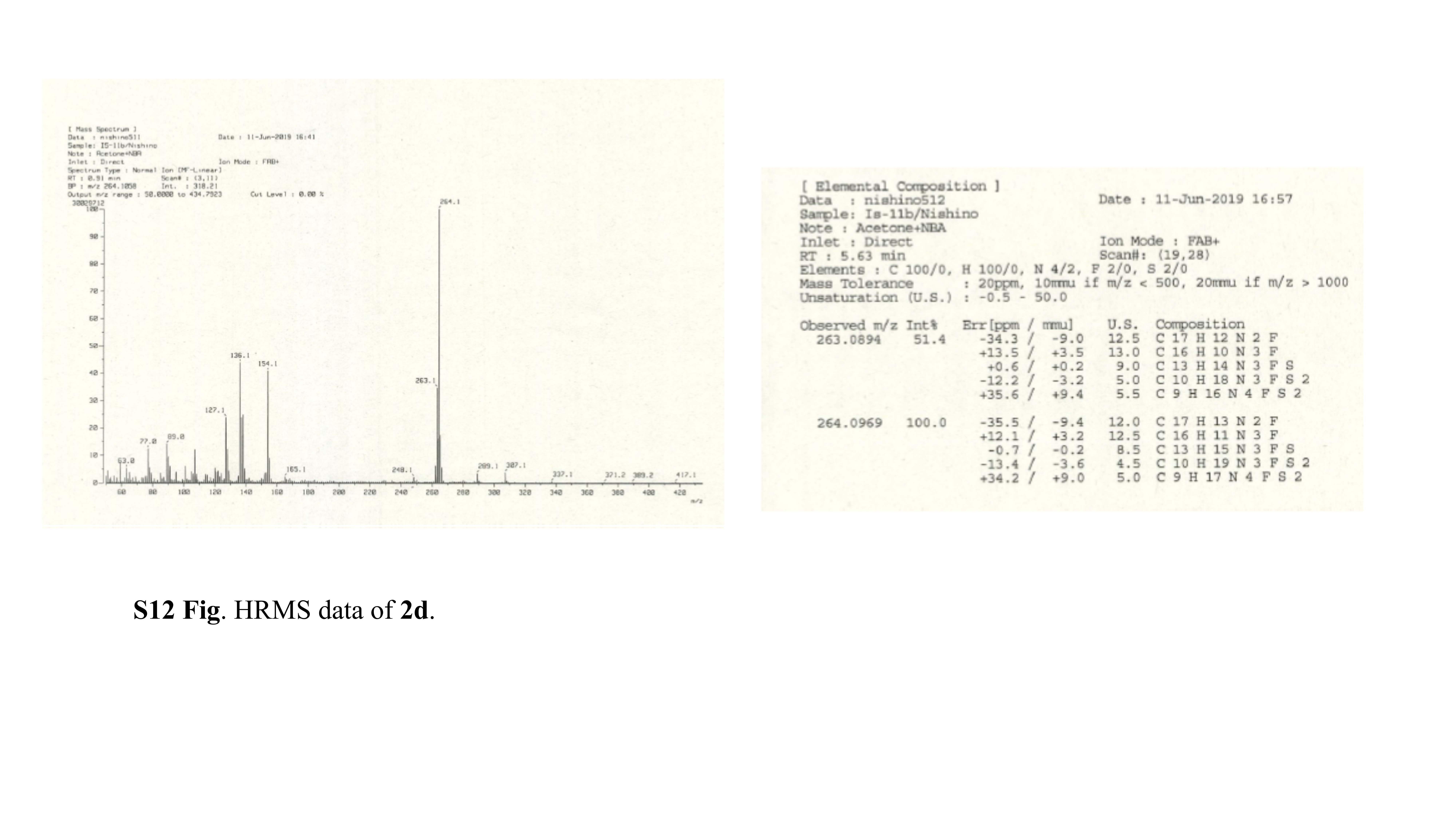

Supplement: S12 Fig — (TIF) [file pone.0318999.s012.tif]

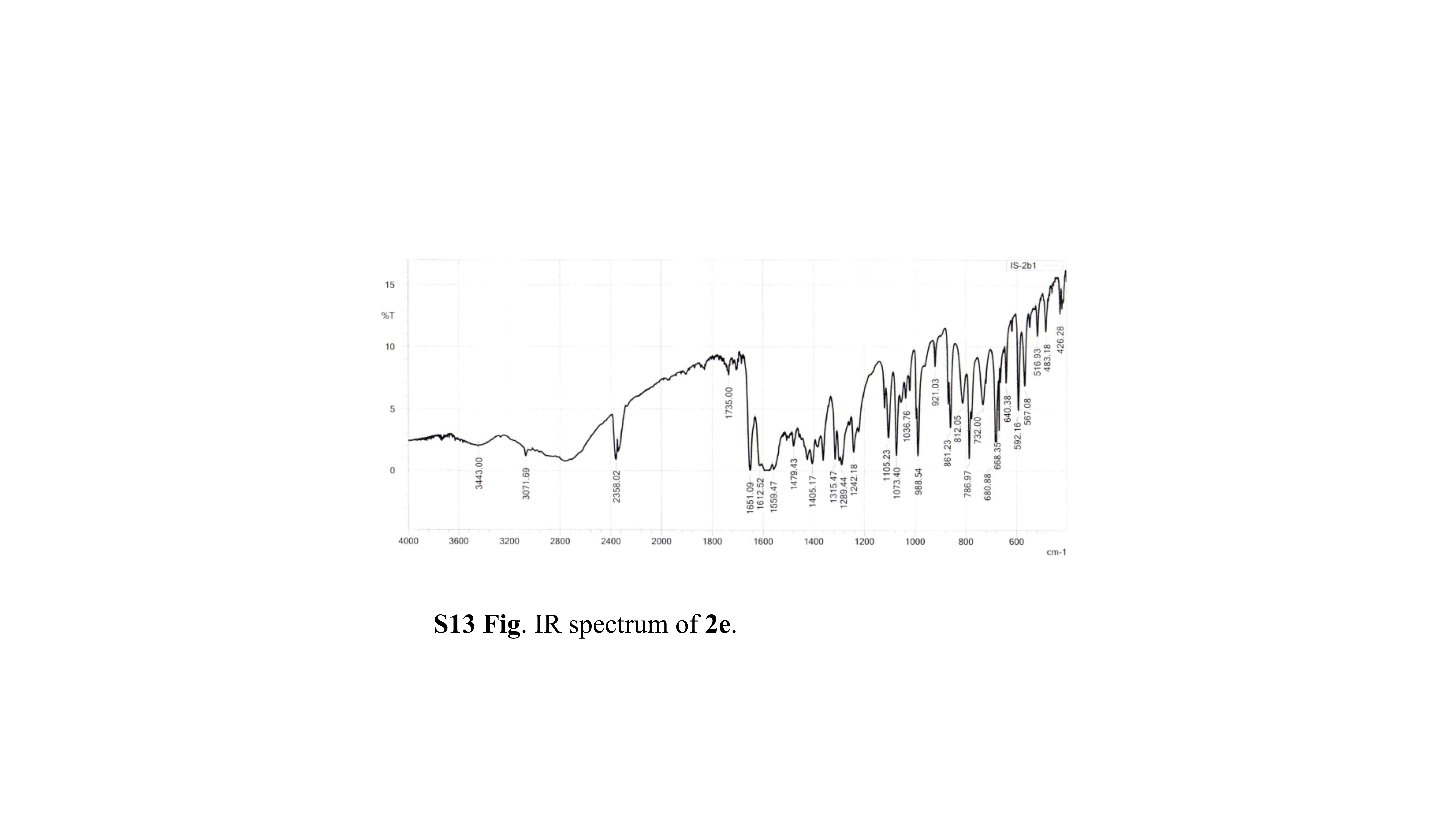

Supplement: S13 Fig — (TIF) [file pone.0318999.s013.tif]

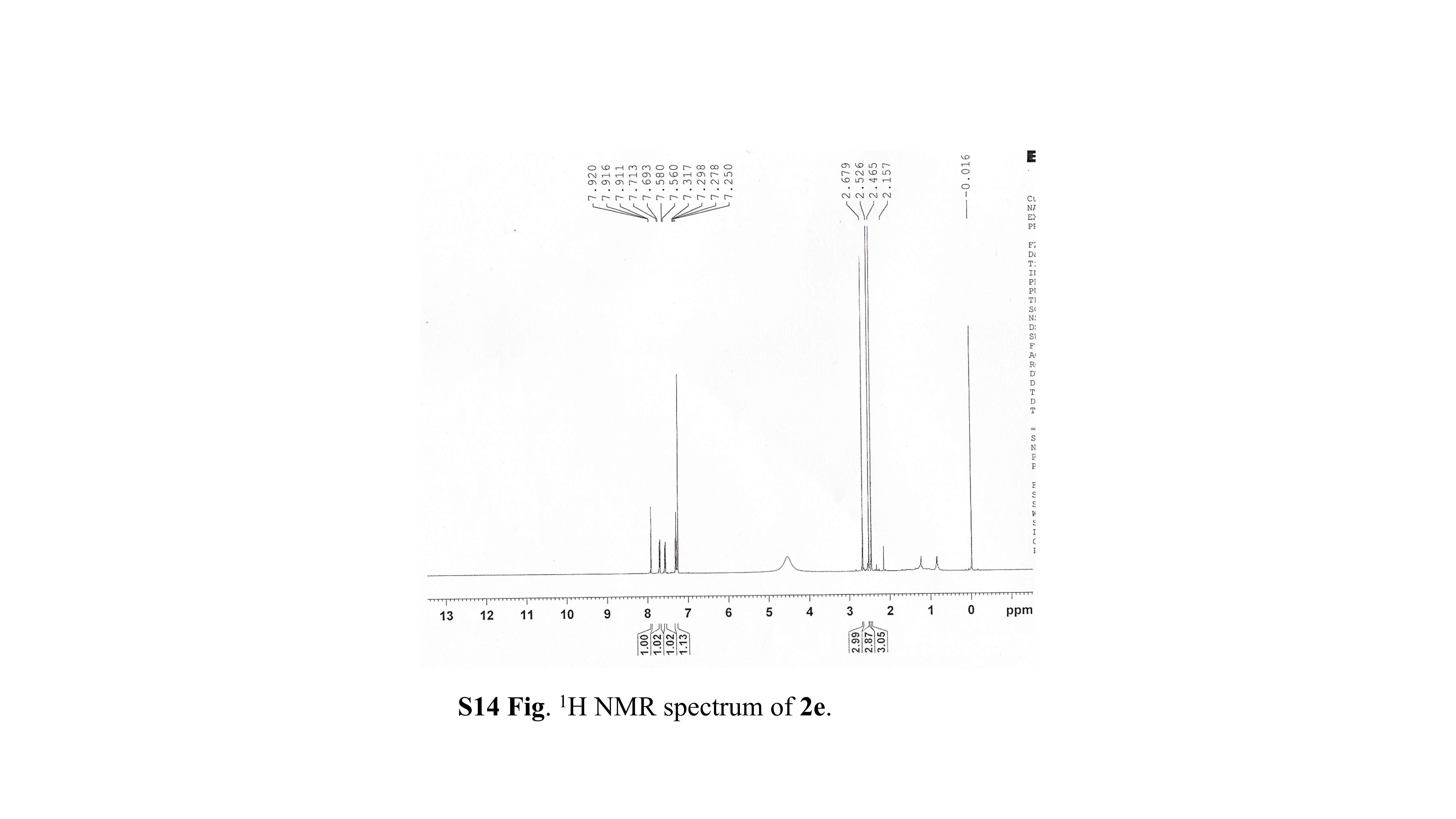

Supplement: S14 Fig — 1H NMR spectrum of 2e. (TIF) [file pone.0318999.s014.tif]

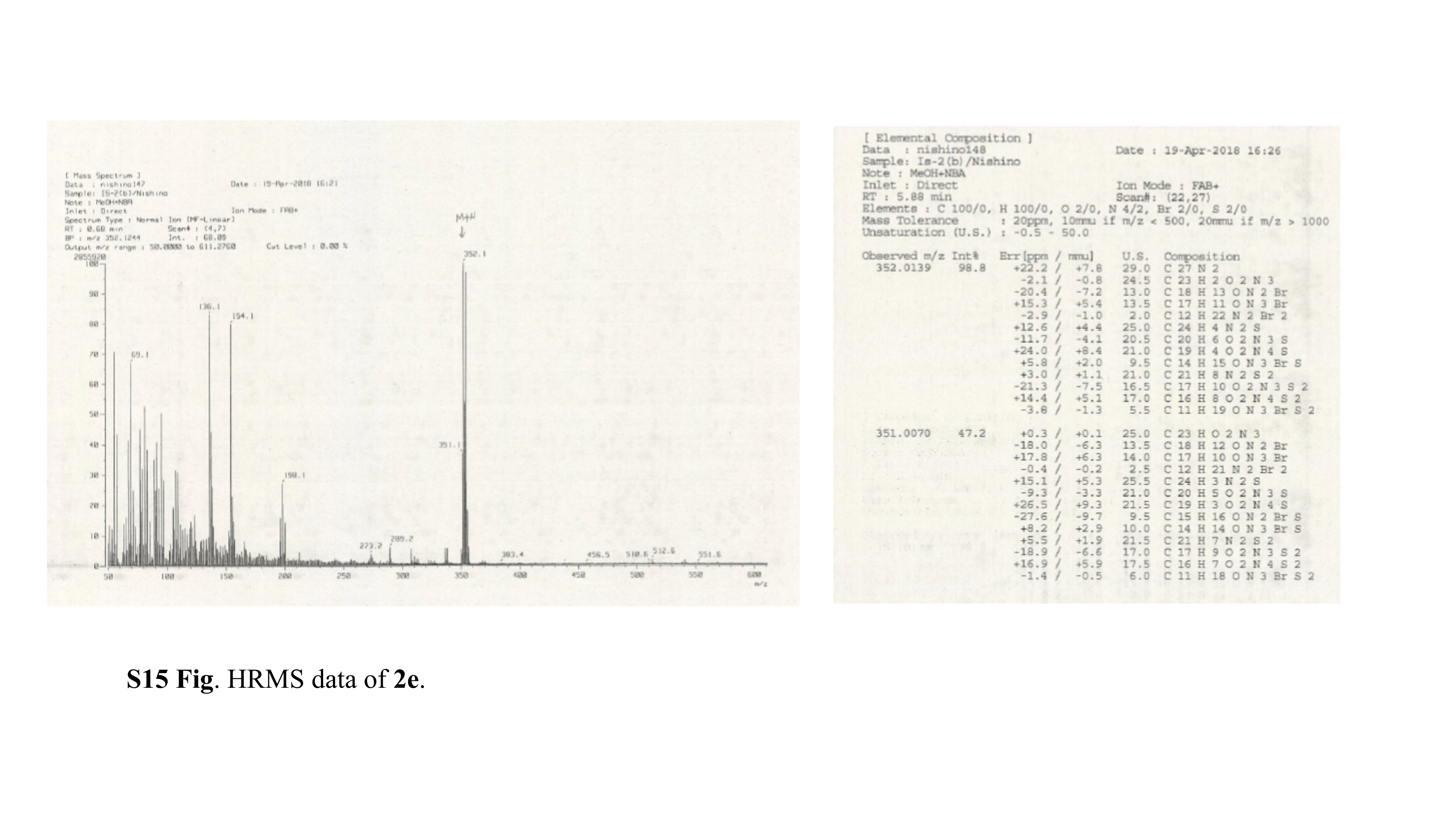

Supplement: S15 Fig — (TIF) [file pone.0318999.s015.tif]

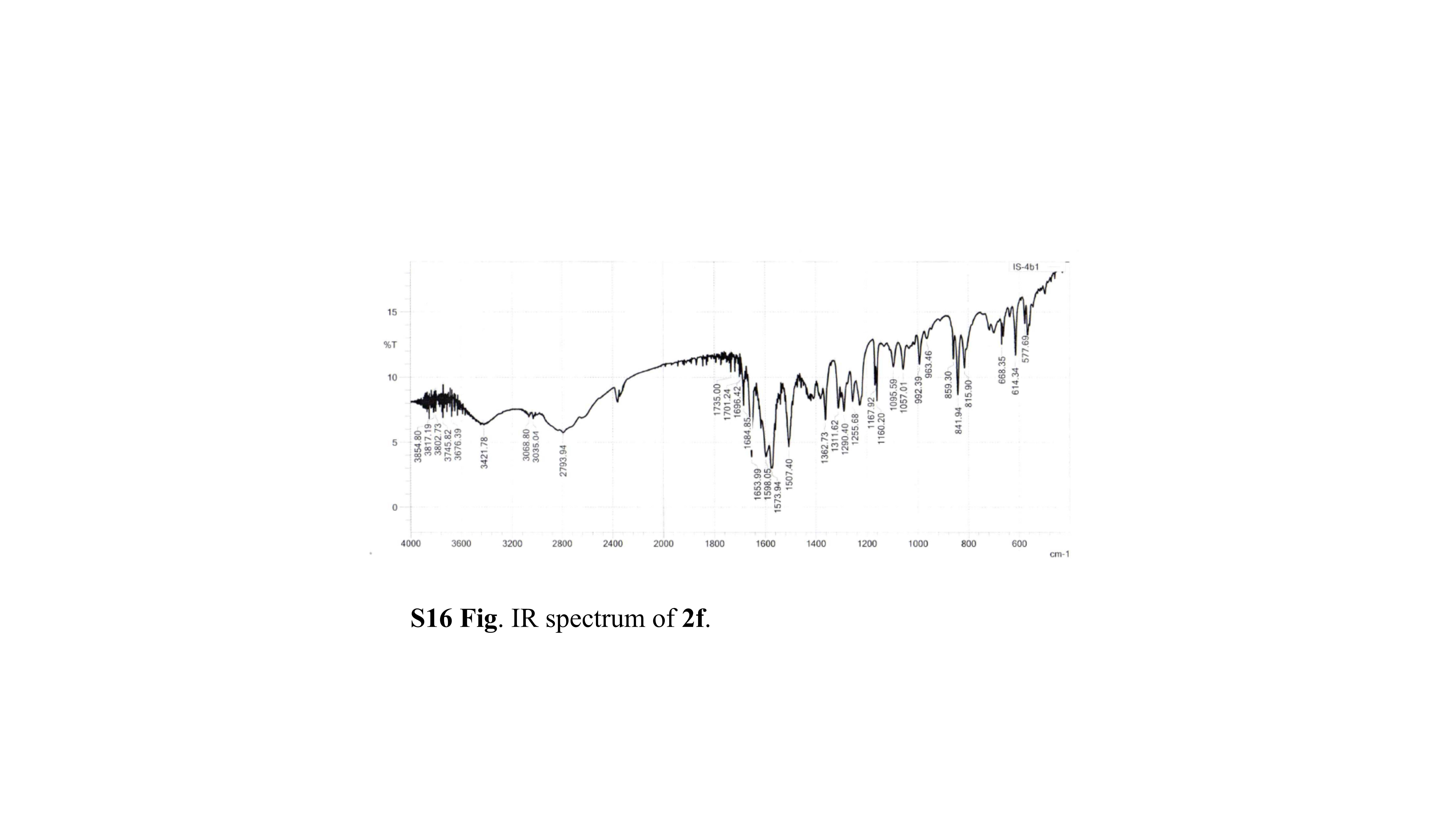

Supplement: S16 Fig — (TIF) [file pone.0318999.s016.tif]

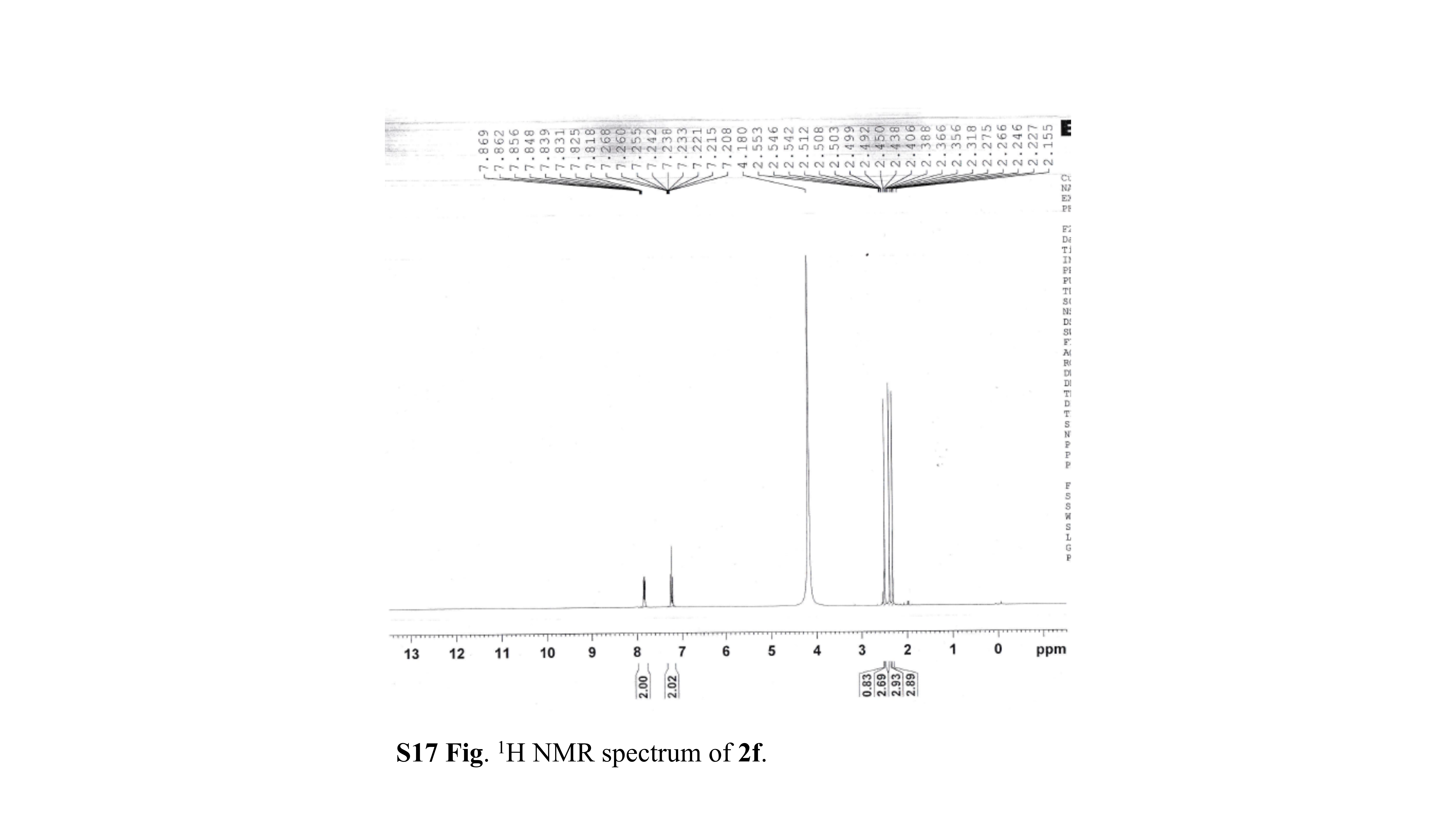

Supplement: S17 Fig — 1H NMR spectrum of 2f. (TIF) [file pone.0318999.s017.tif]

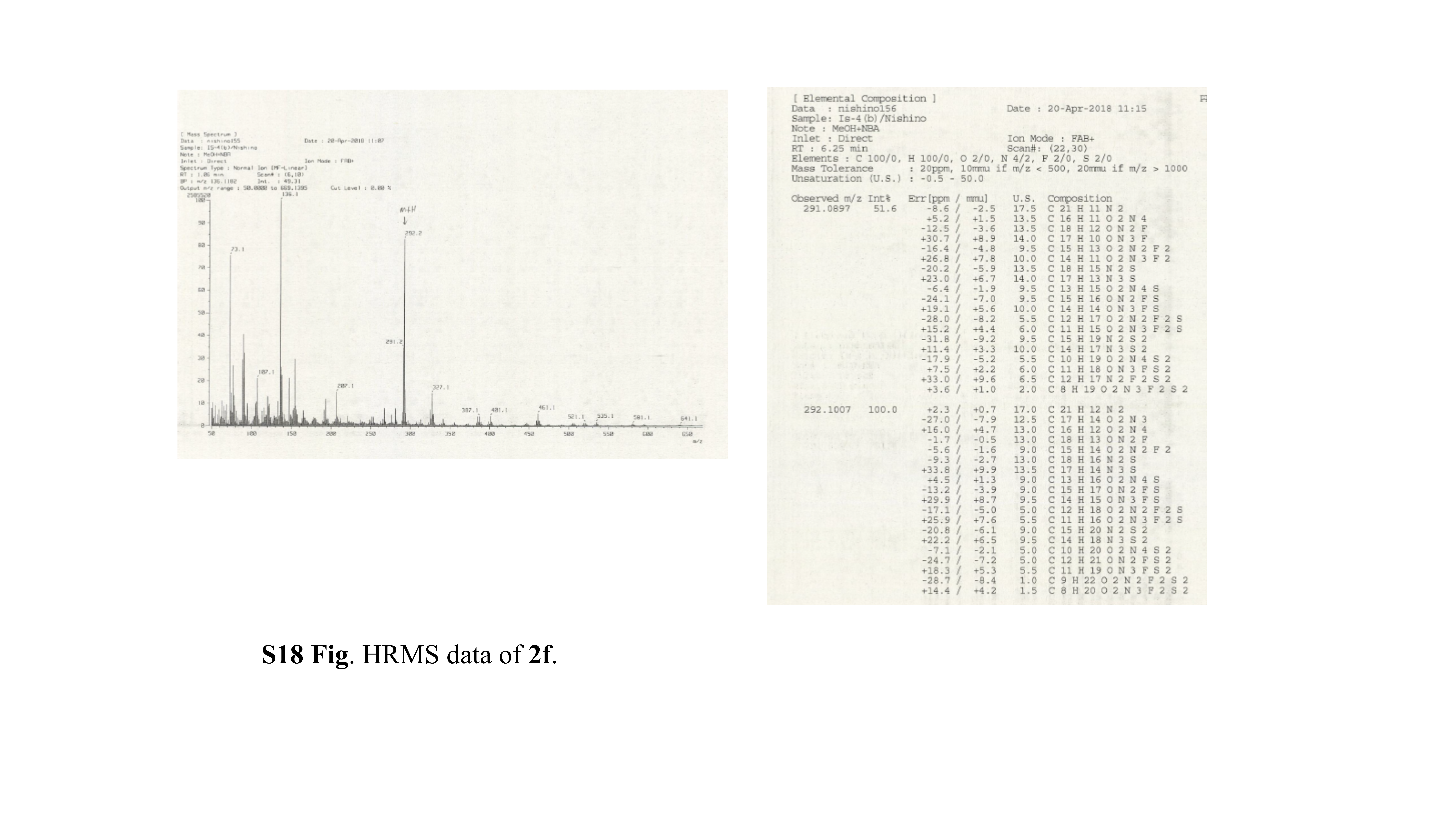

Supplement: S18 Fig — (TIF) [file pone.0318999.s018.tif]

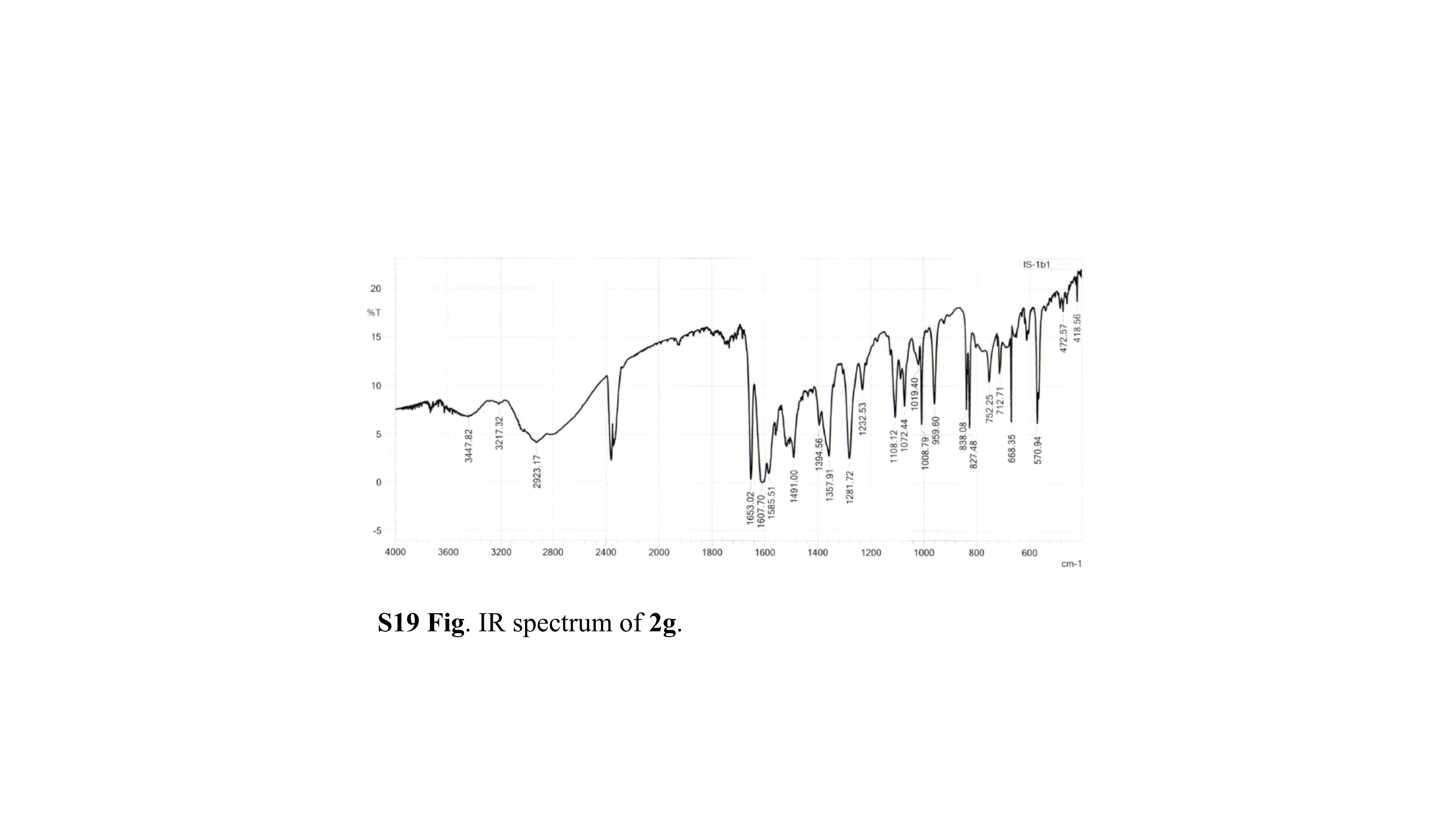

Supplement: S19 Fig — (TIF) [file pone.0318999.s019.tif]

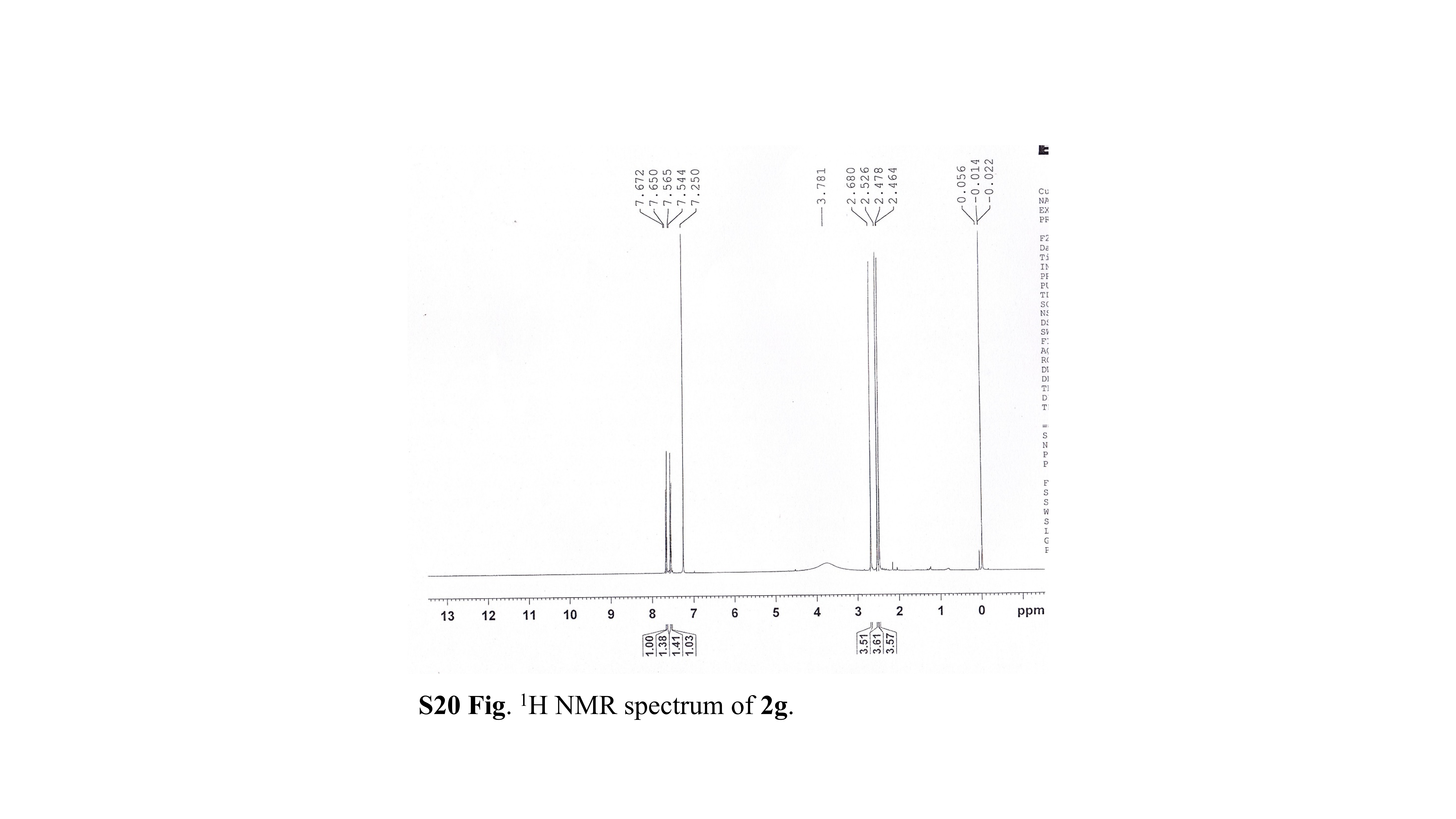

Supplement: S20 Fig — 1H NMR spectrum of 2g. (TIF) [file pone.0318999.s020.tif]

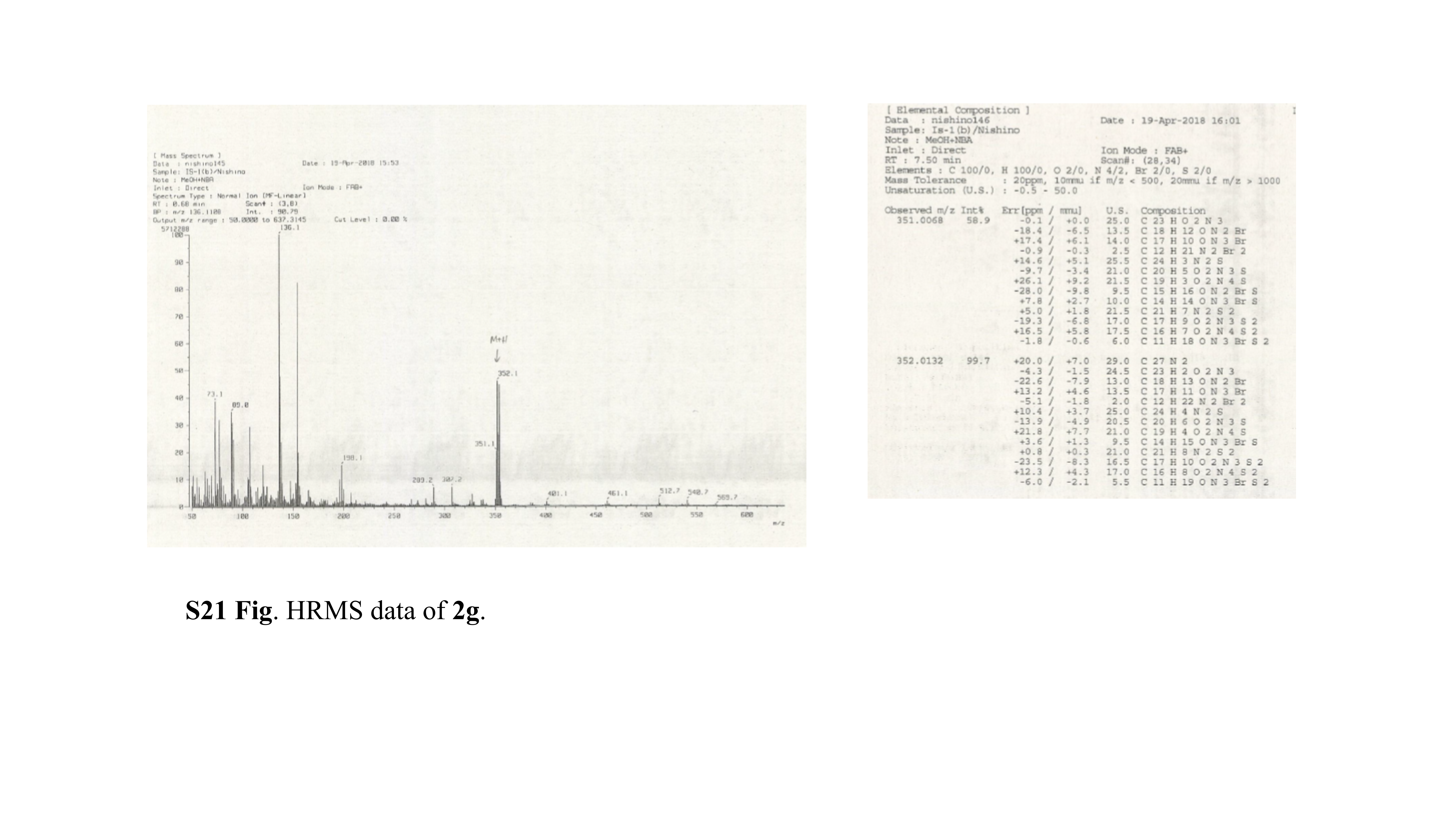

Supplement: S21 Fig — (TIF) [file pone.0318999.s021.tif]

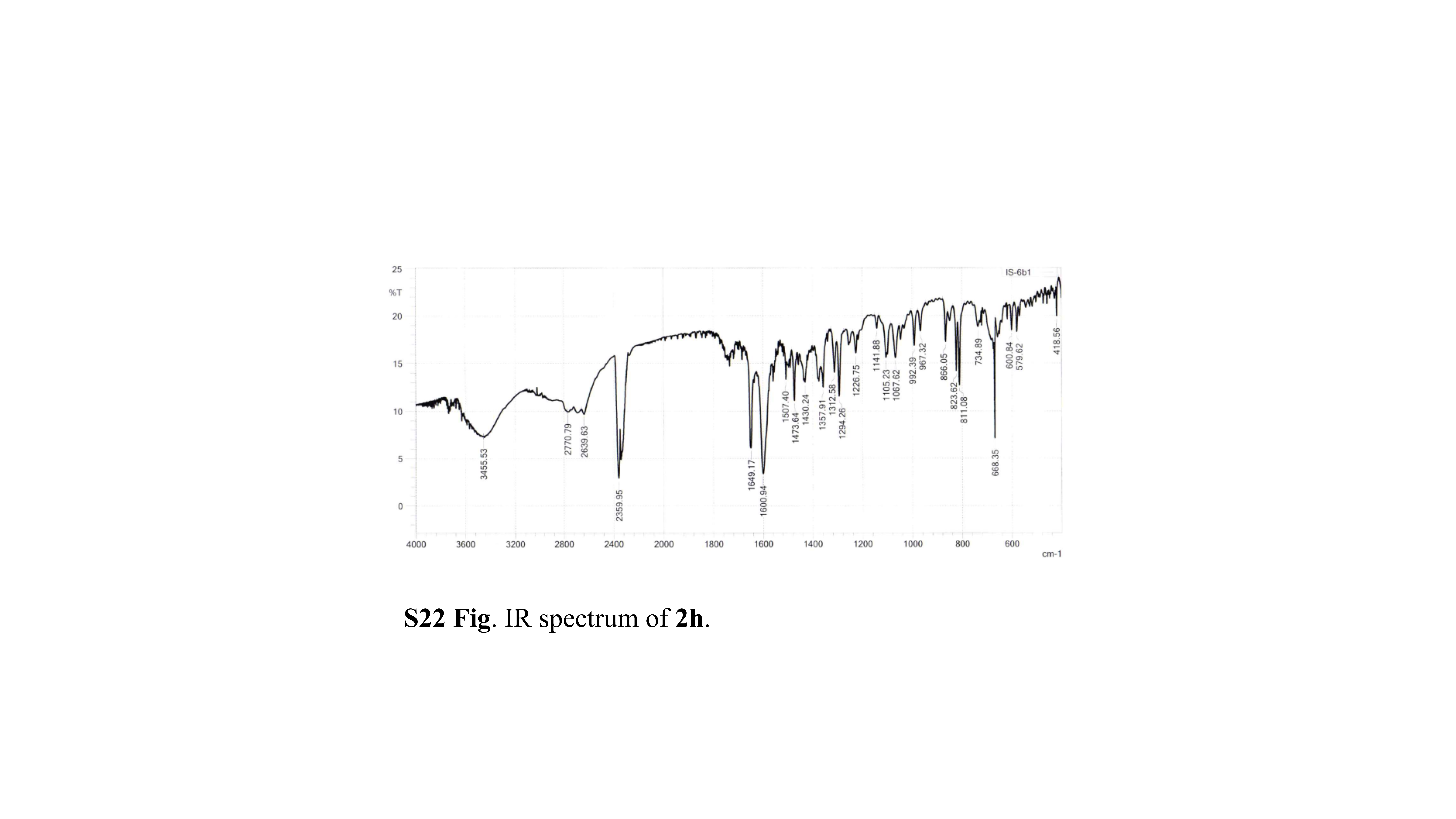

Supplement: S22 Fig — (TIF) [file pone.0318999.s022.tif]

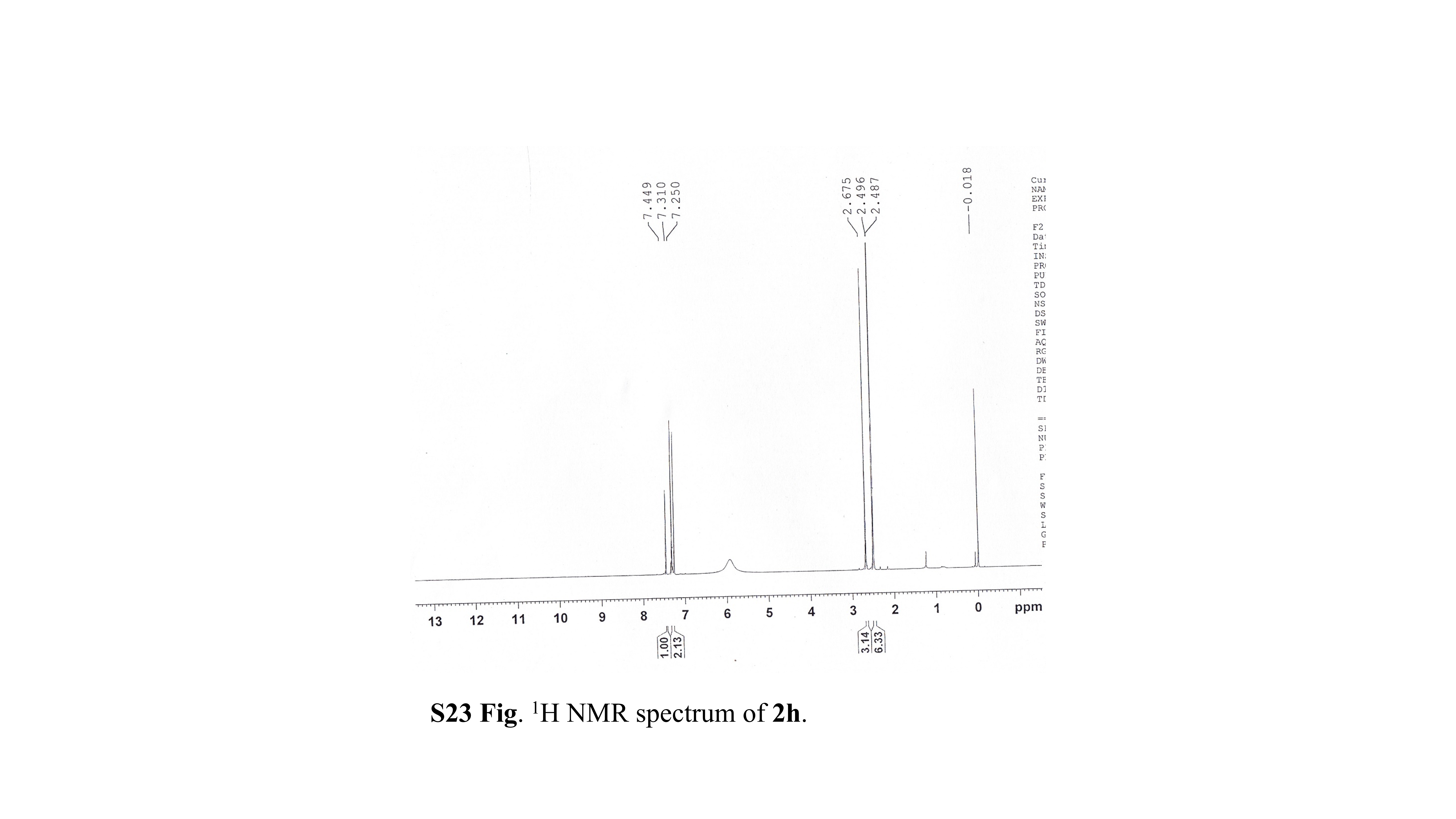

Supplement: S23 Fig — 1H NMR spectrum of 2h. (TIF) [file pone.0318999.s023.tif]

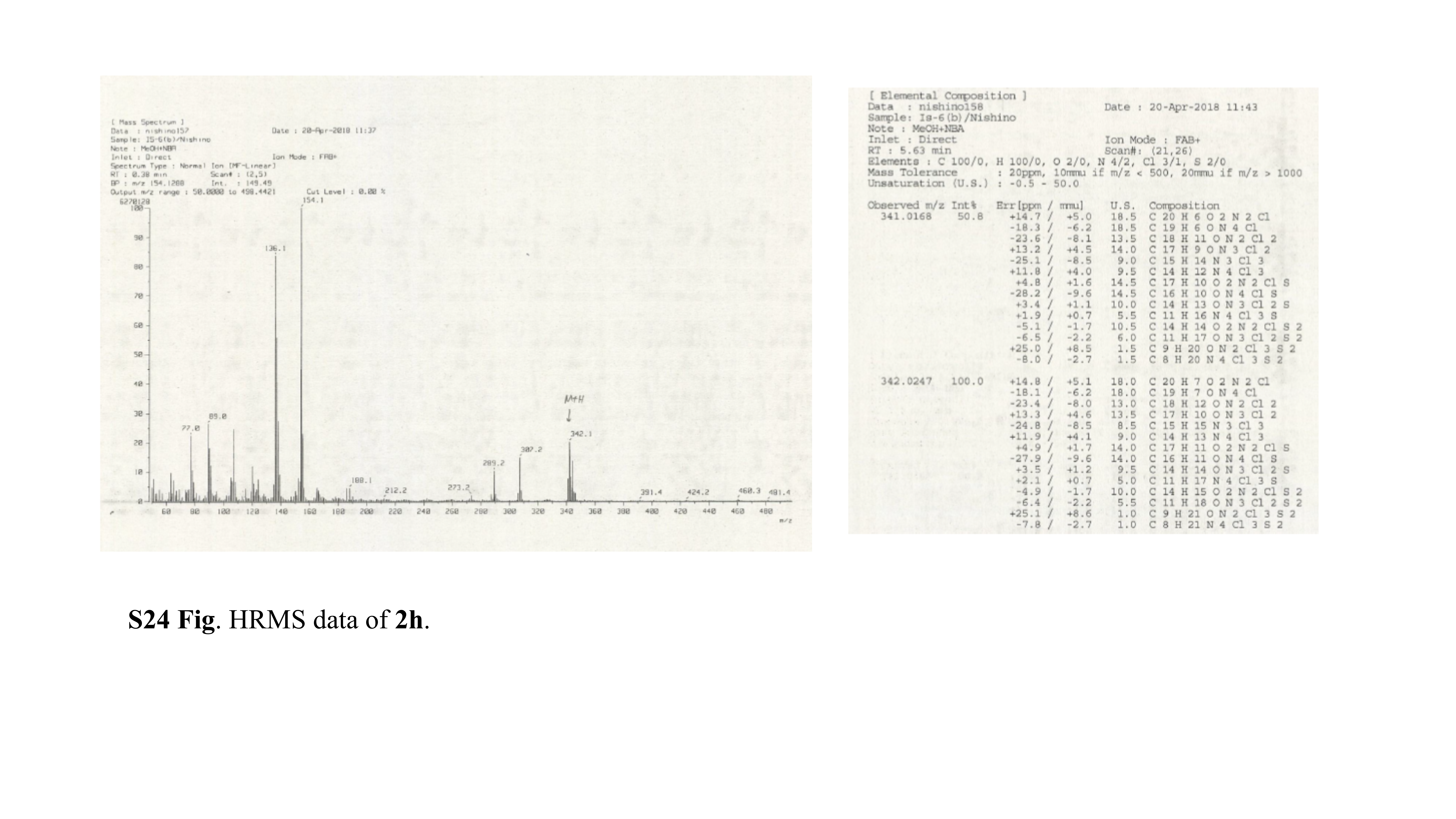

Supplement: S24 Fig — (TIF) [file pone.0318999.s024.tif]

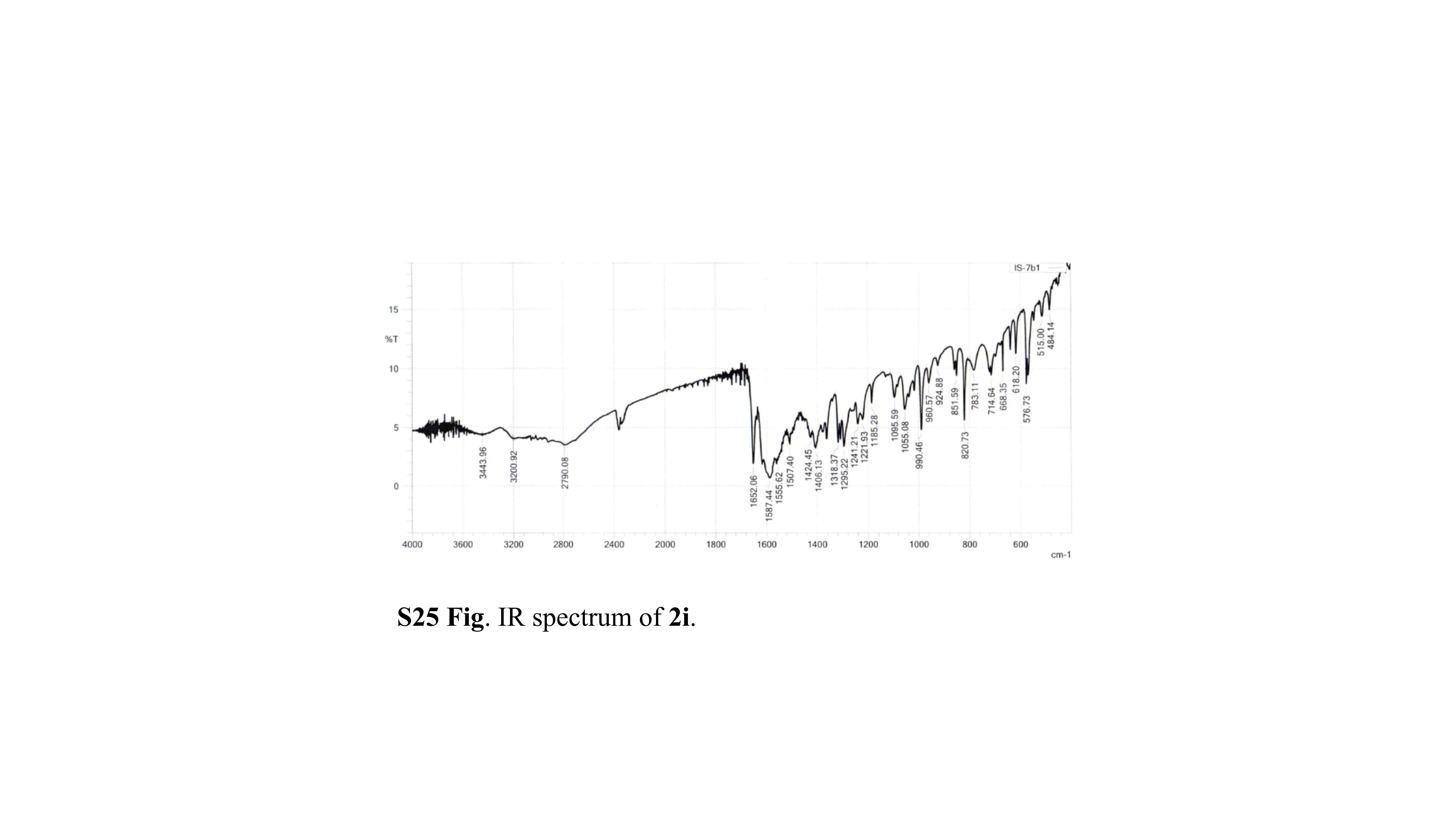

Supplement: S25 Fig — (TIF) [file pone.0318999.s025.tif]

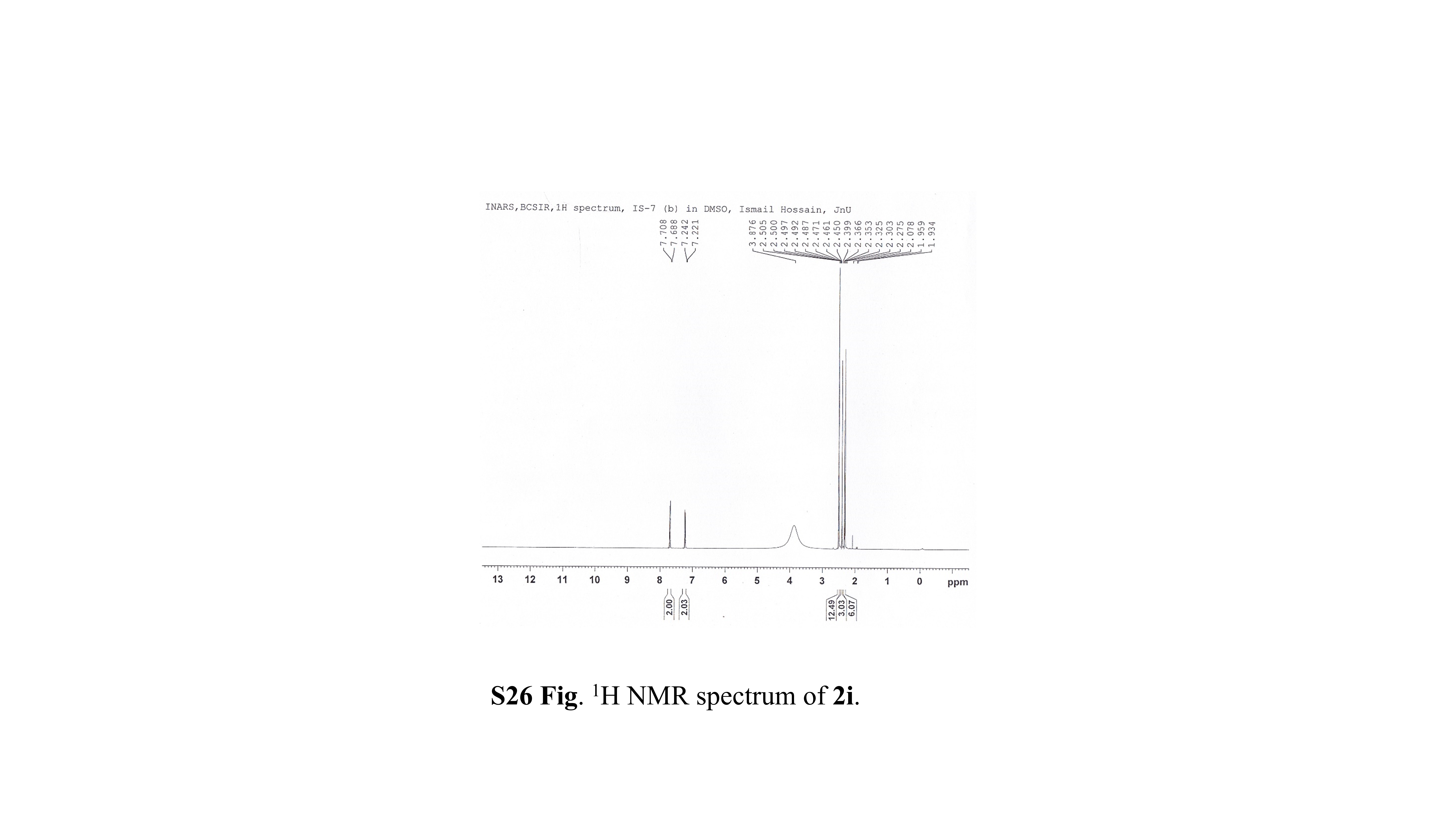

Supplement: S26 Fig — 1H NMR spectrum of 2i. (TIF) [file pone.0318999.s026.tif]

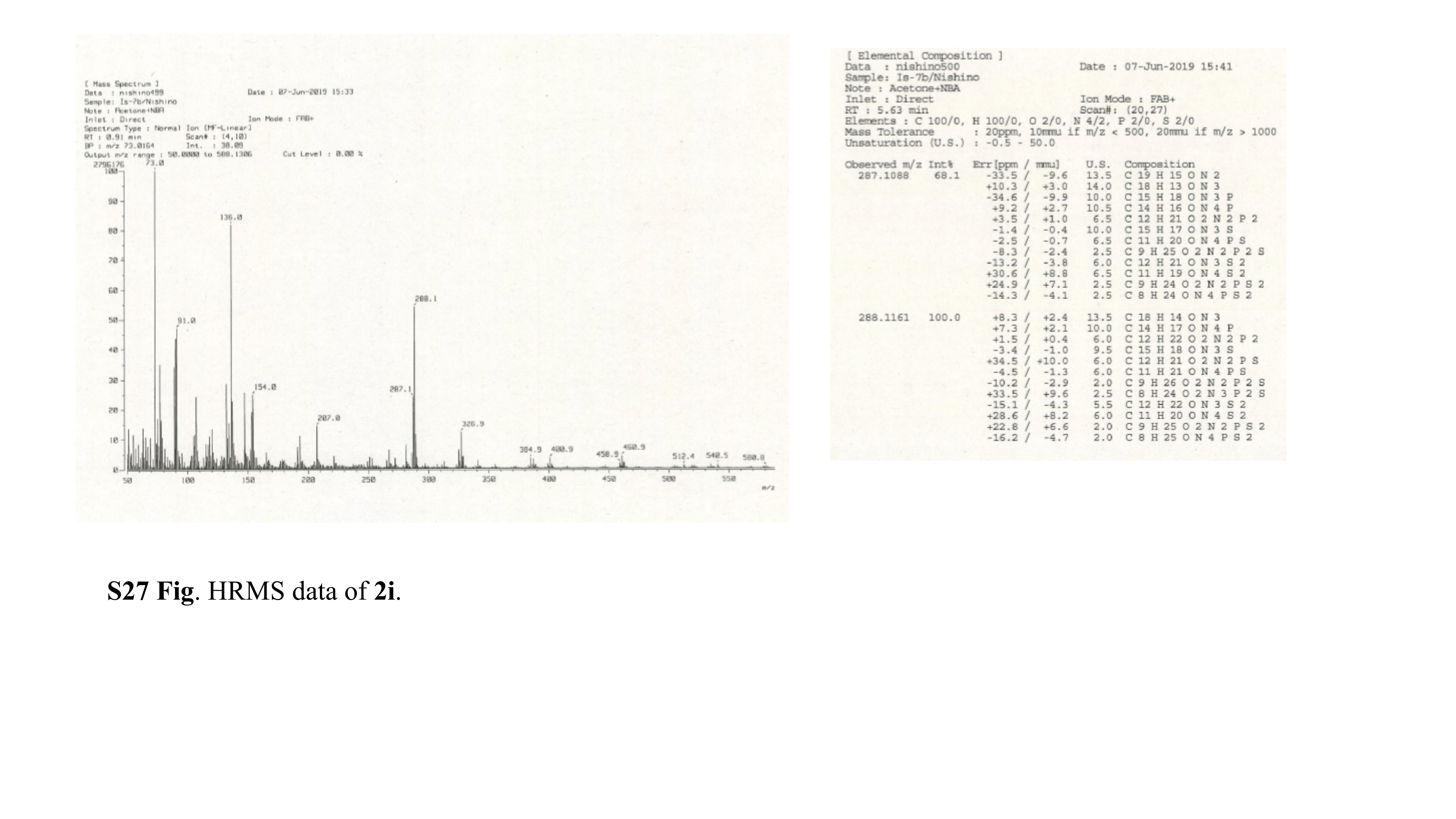

Supplement: S27 Fig — (TIF) [file pone.0318999.s027.tif]

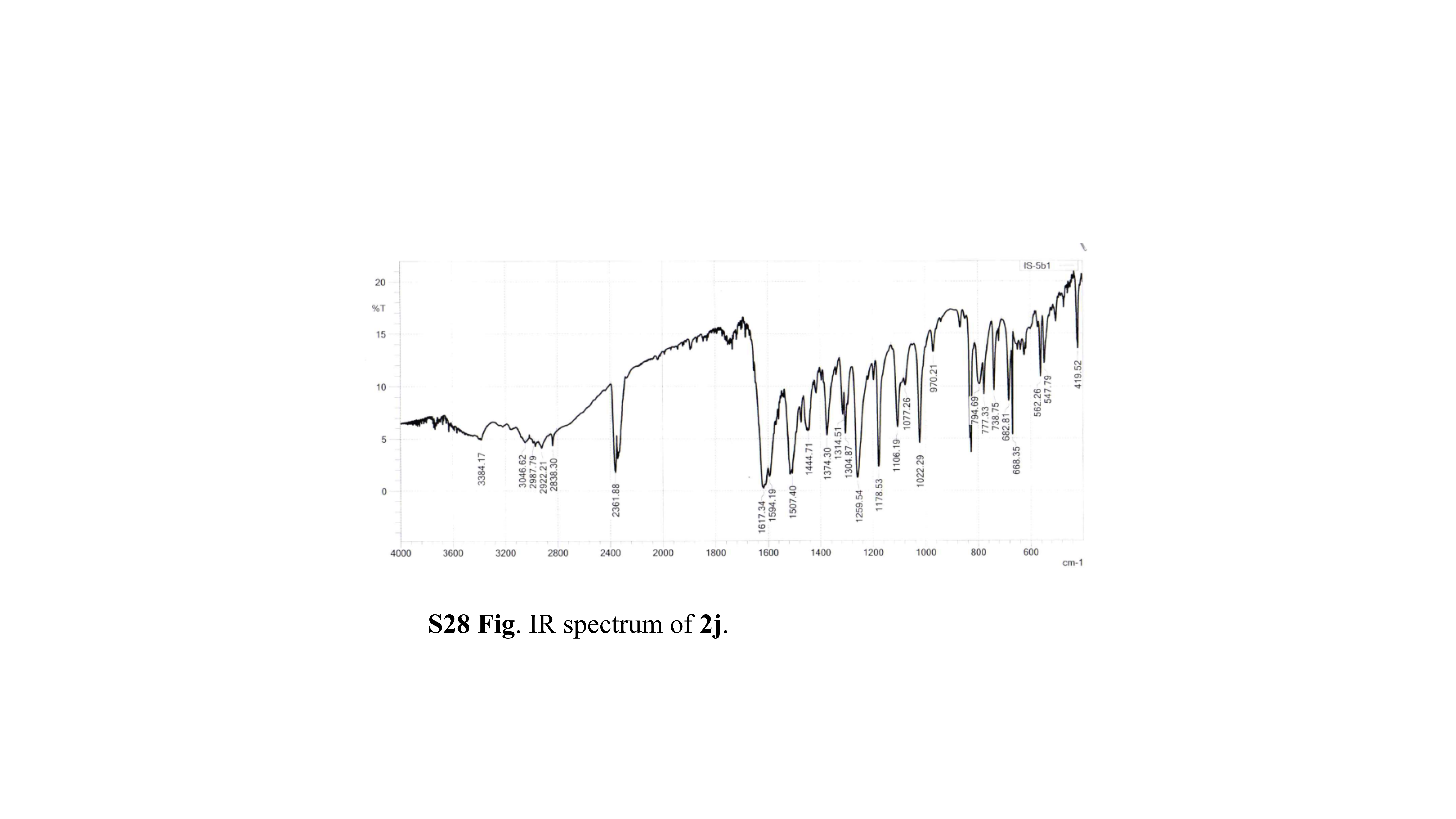

Supplement: S28 Fig — (TIF) [file pone.0318999.s028.tif]

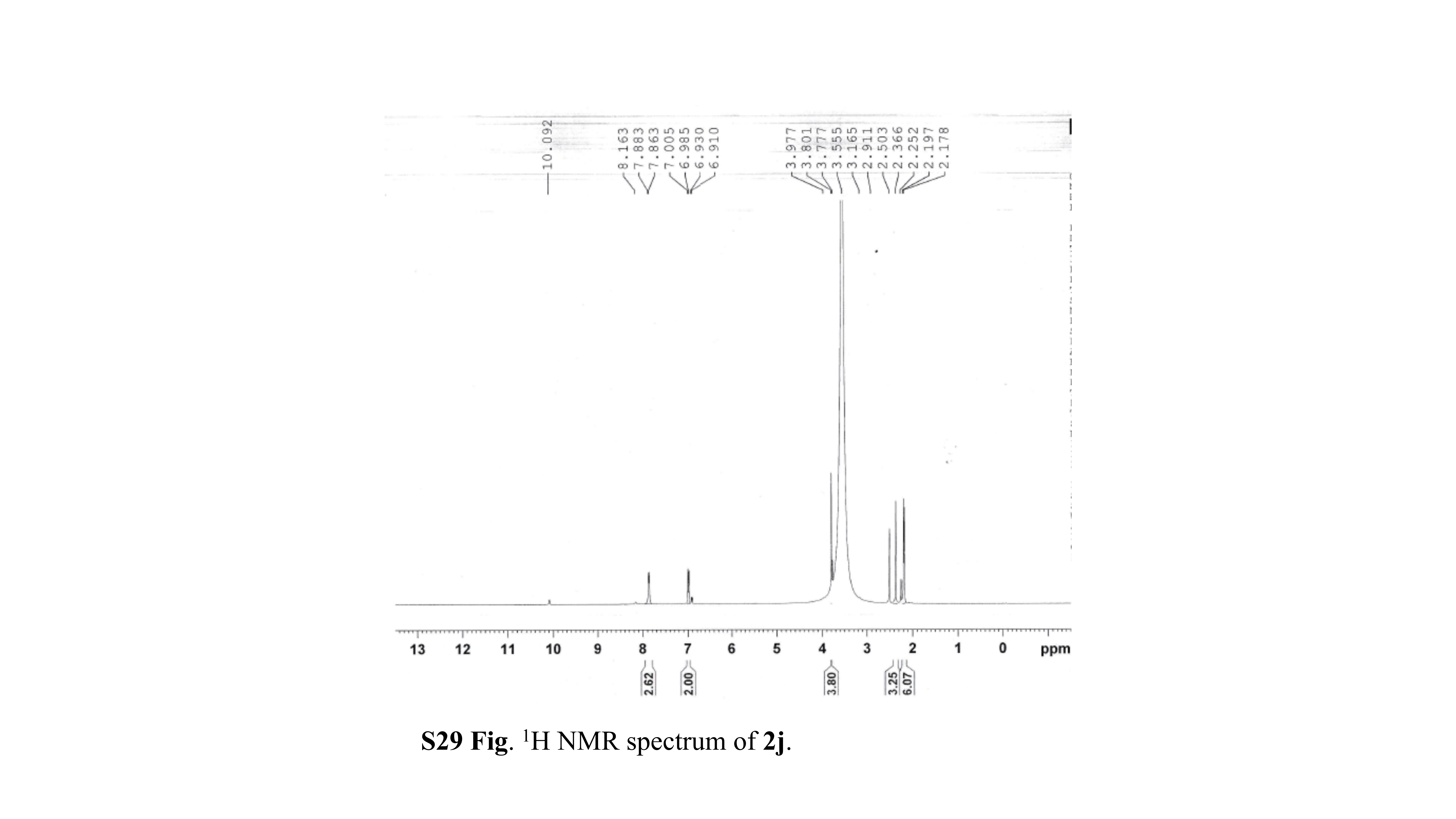

Supplement: S29 Fig — 1H NMR spectrum of 2j. (TIF) [file pone.0318999.s029.tif]

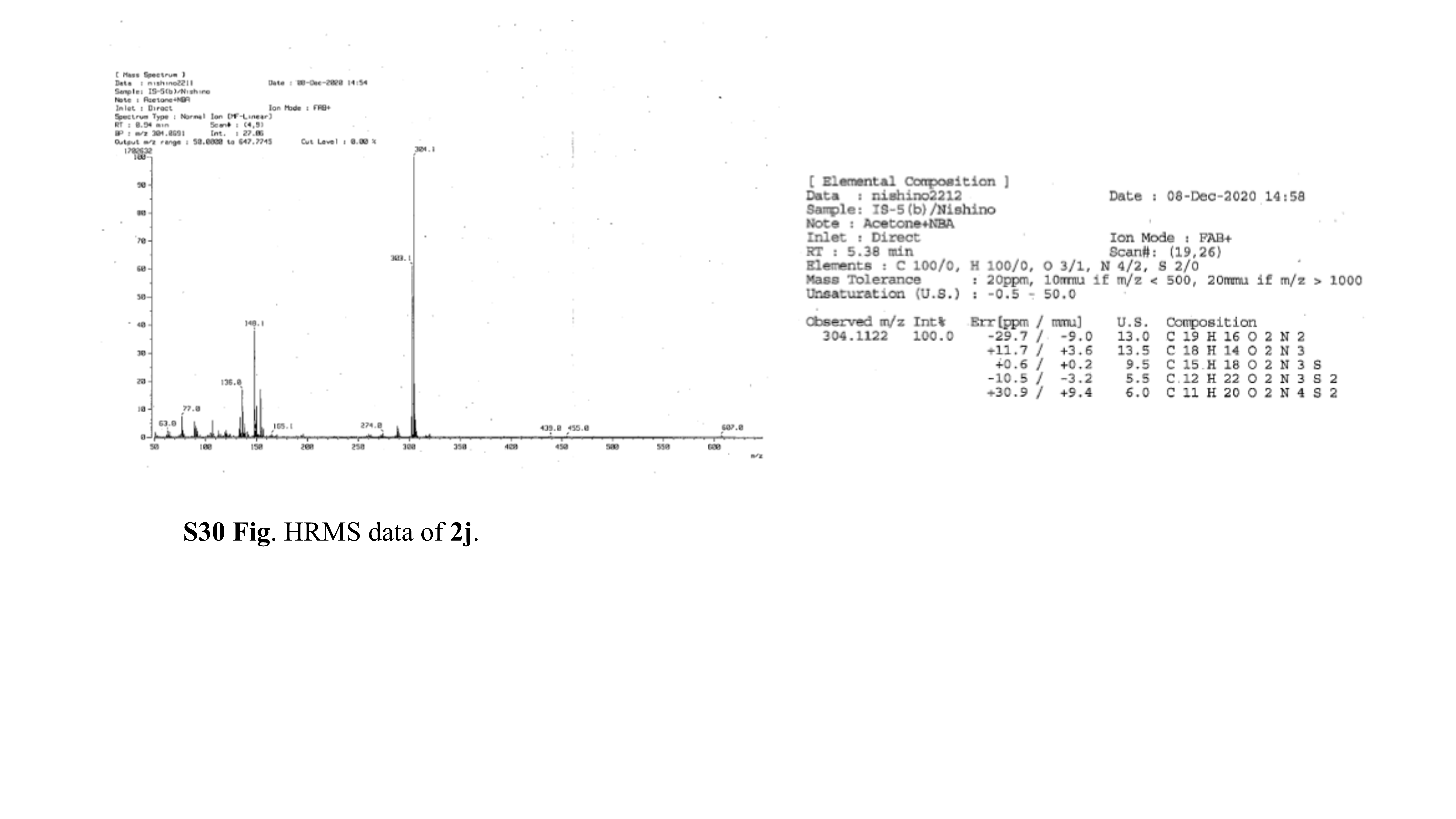

Supplement: S30 Fig — (TIF) [file pone.0318999.s030.tif]

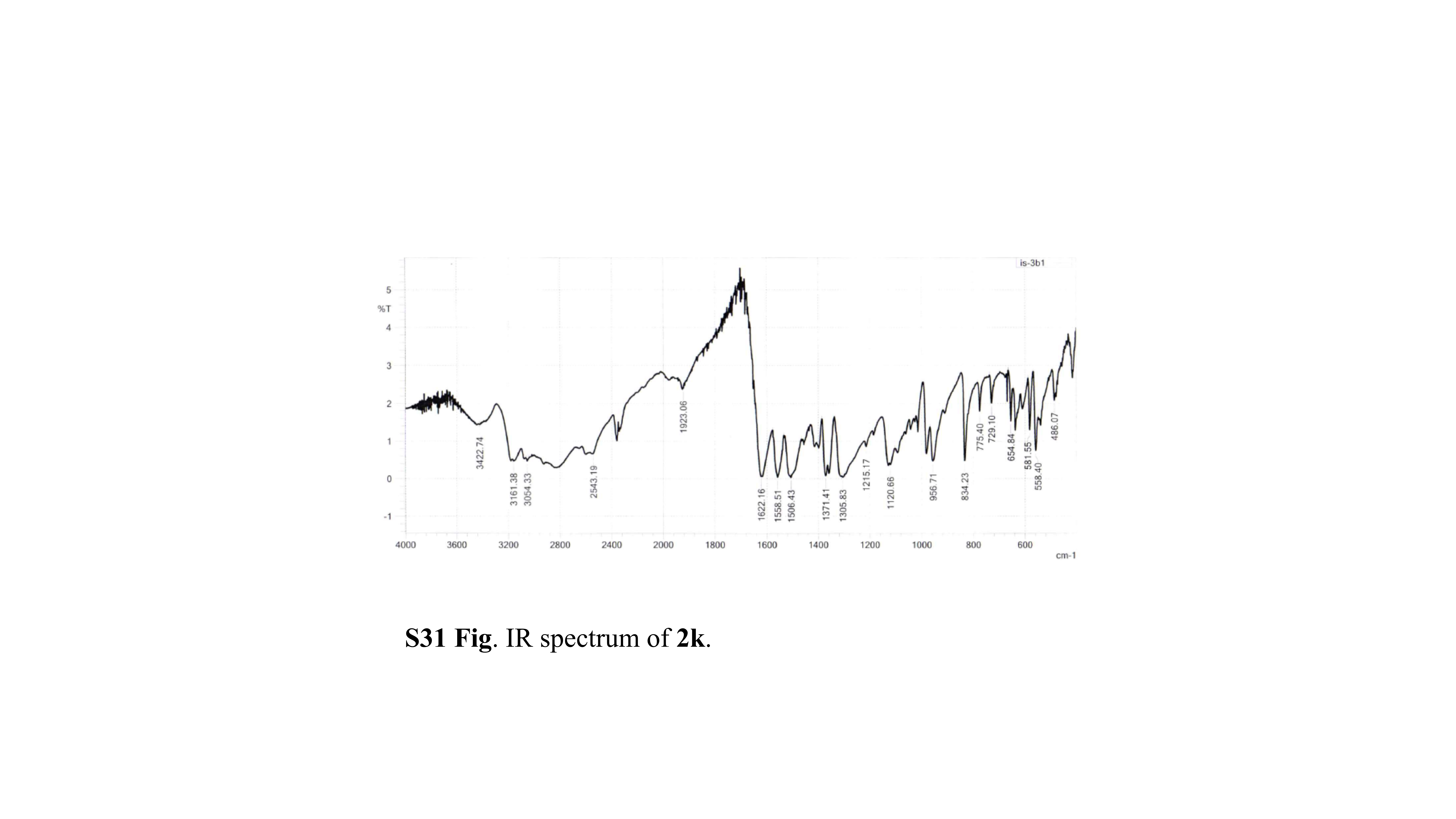

Supplement: S31 Fig — (TIF) [file pone.0318999.s031.tif]

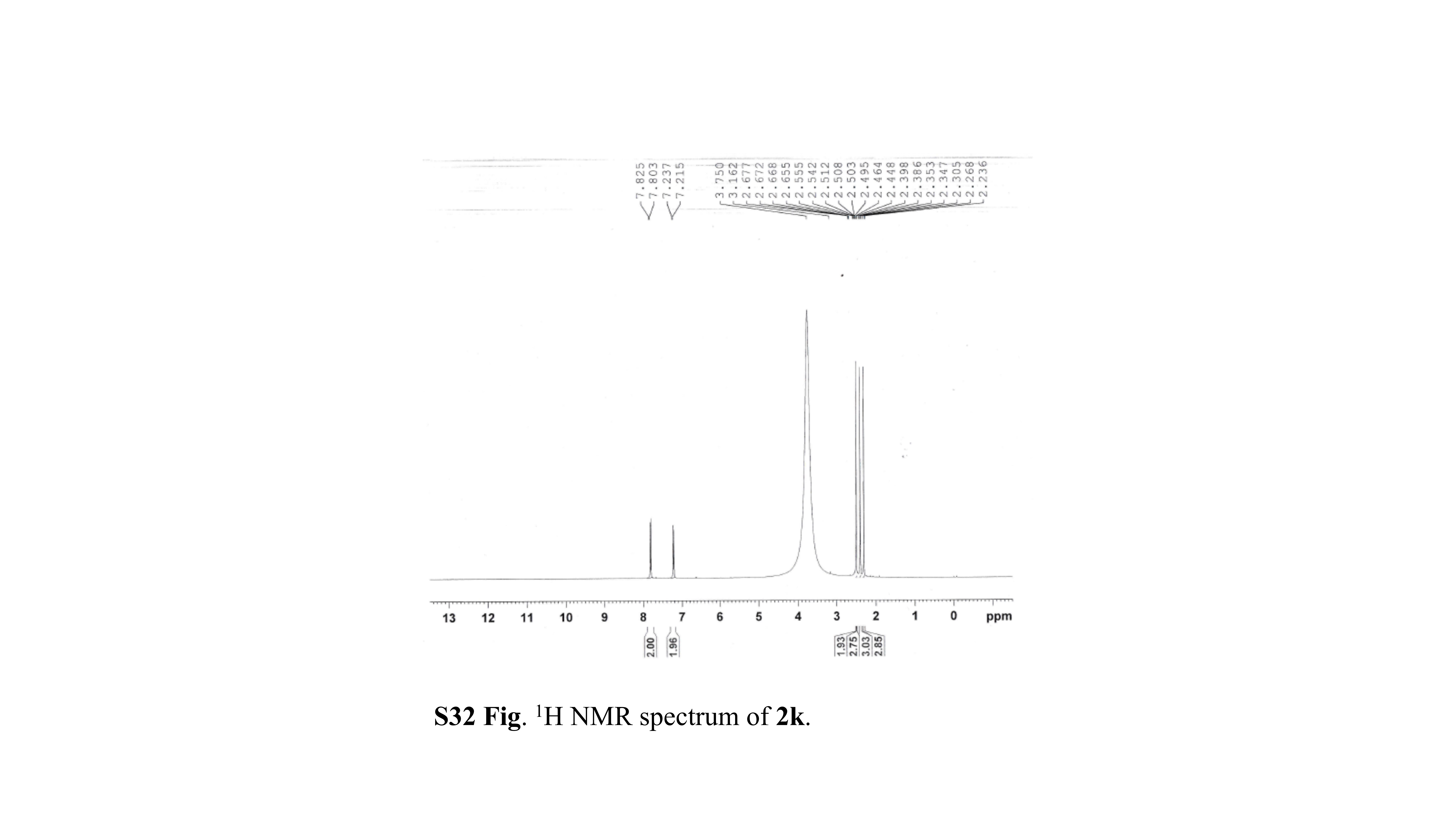

Supplement: S32 Fig — 1H NMR spectrum of 2k. (TIF) [file pone.0318999.s032.tif]

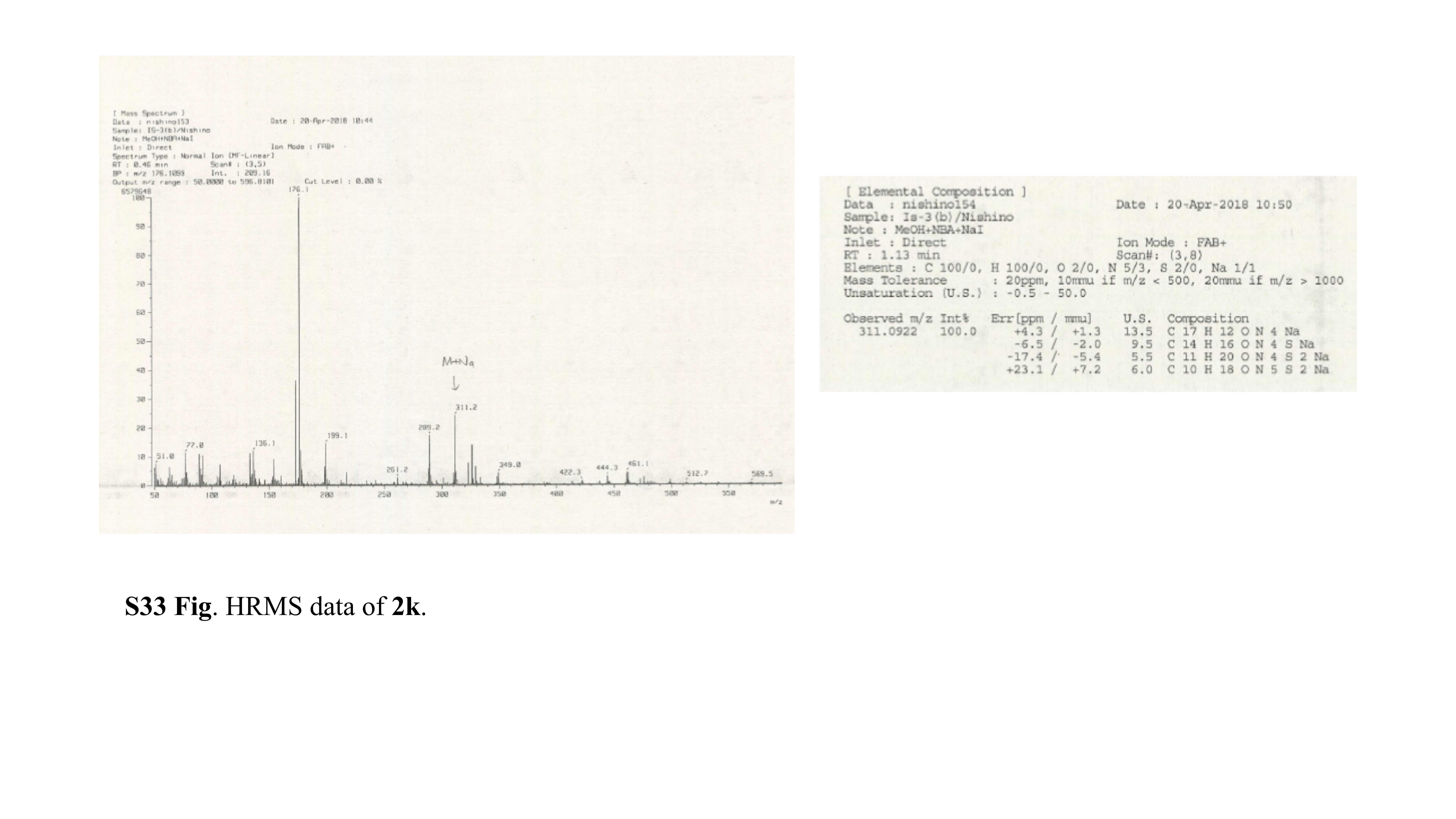

Supplement: S33 Fig — (TIF) [file pone.0318999.s033.tif]

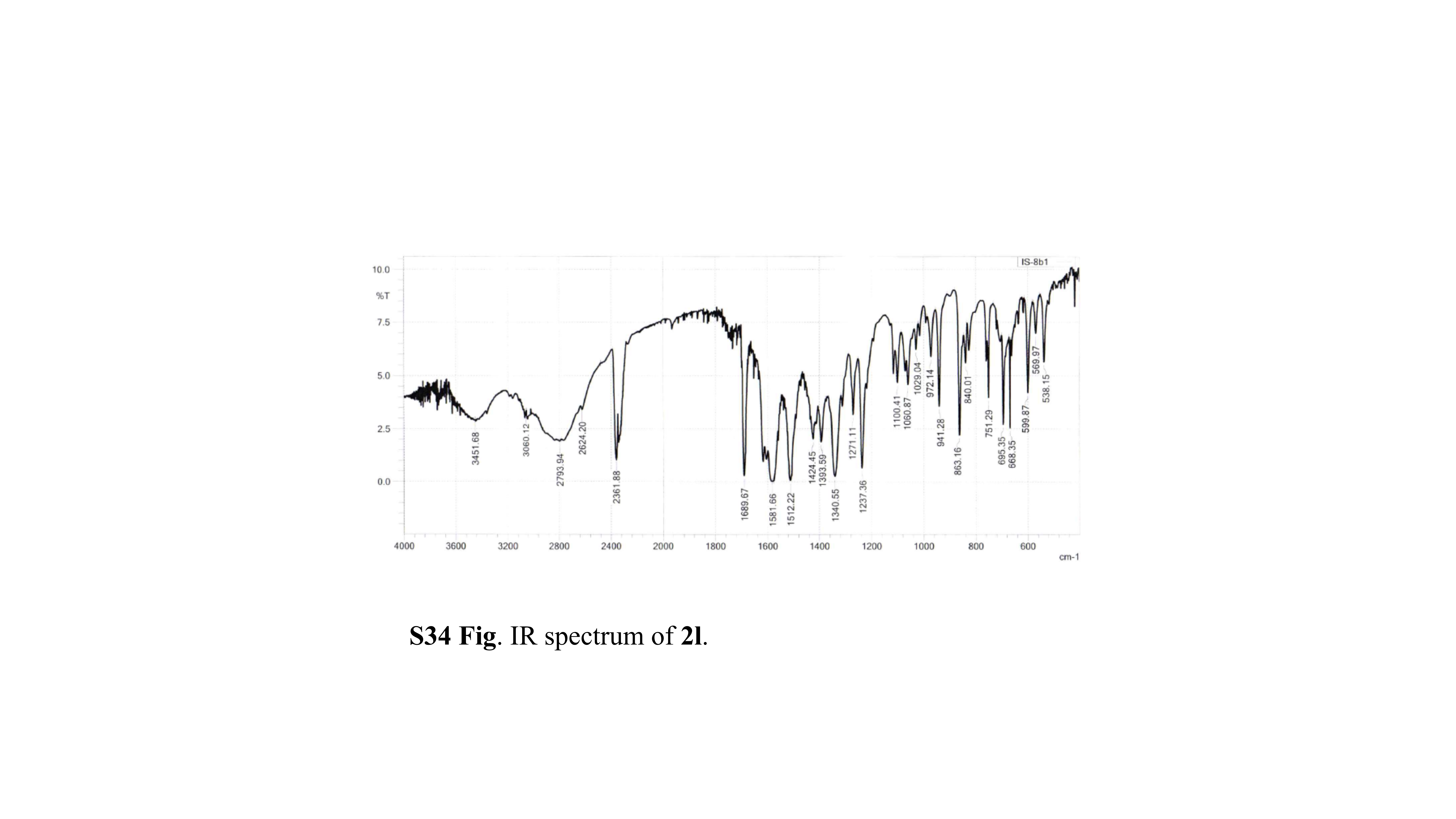

Supplement: S34 Fig — (TIF) [file pone.0318999.s034.tif]

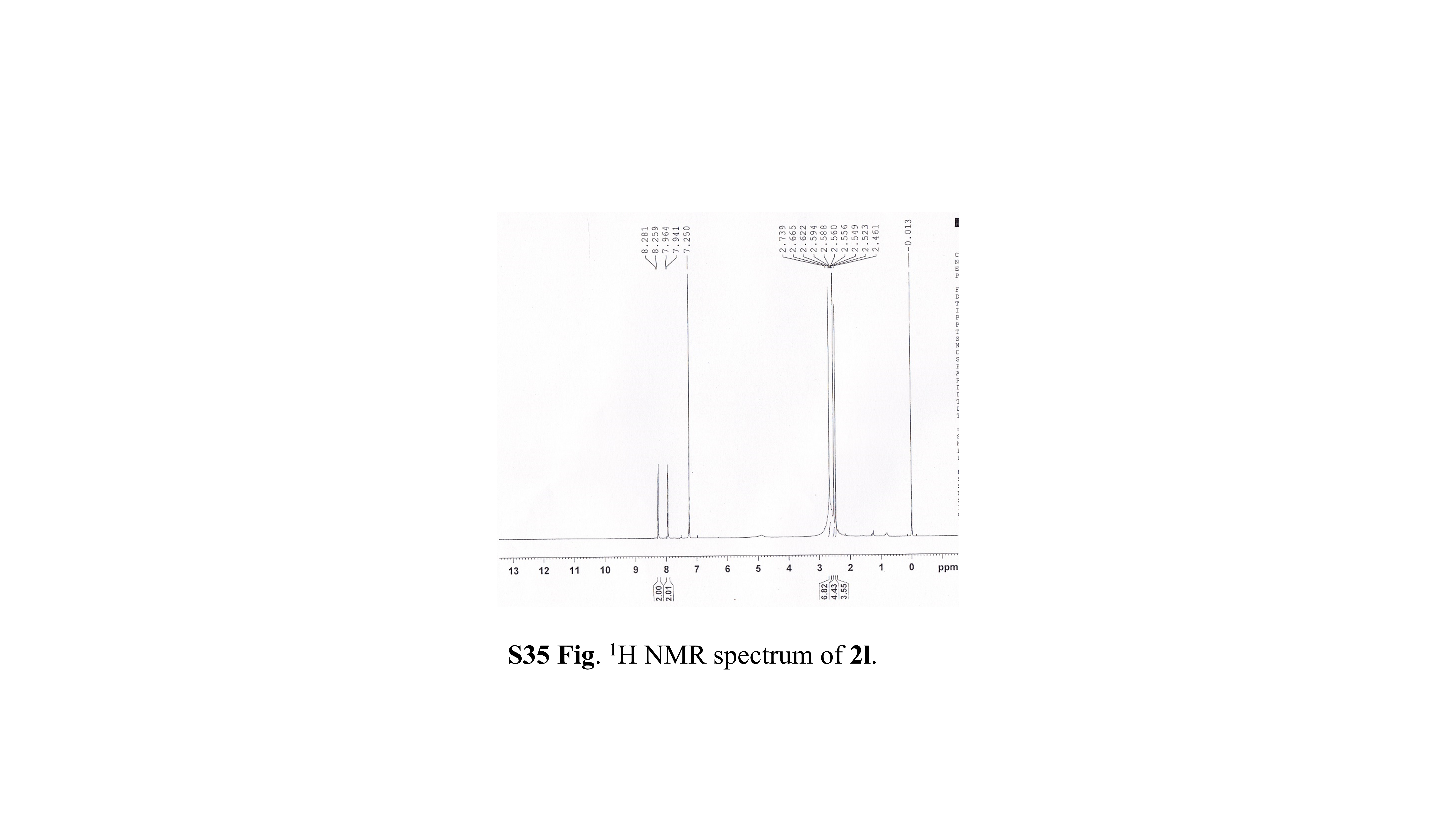

Supplement: S35 Fig — 1H NMR spectrum of 2l. (TIF) [file pone.0318999.s035.tif]

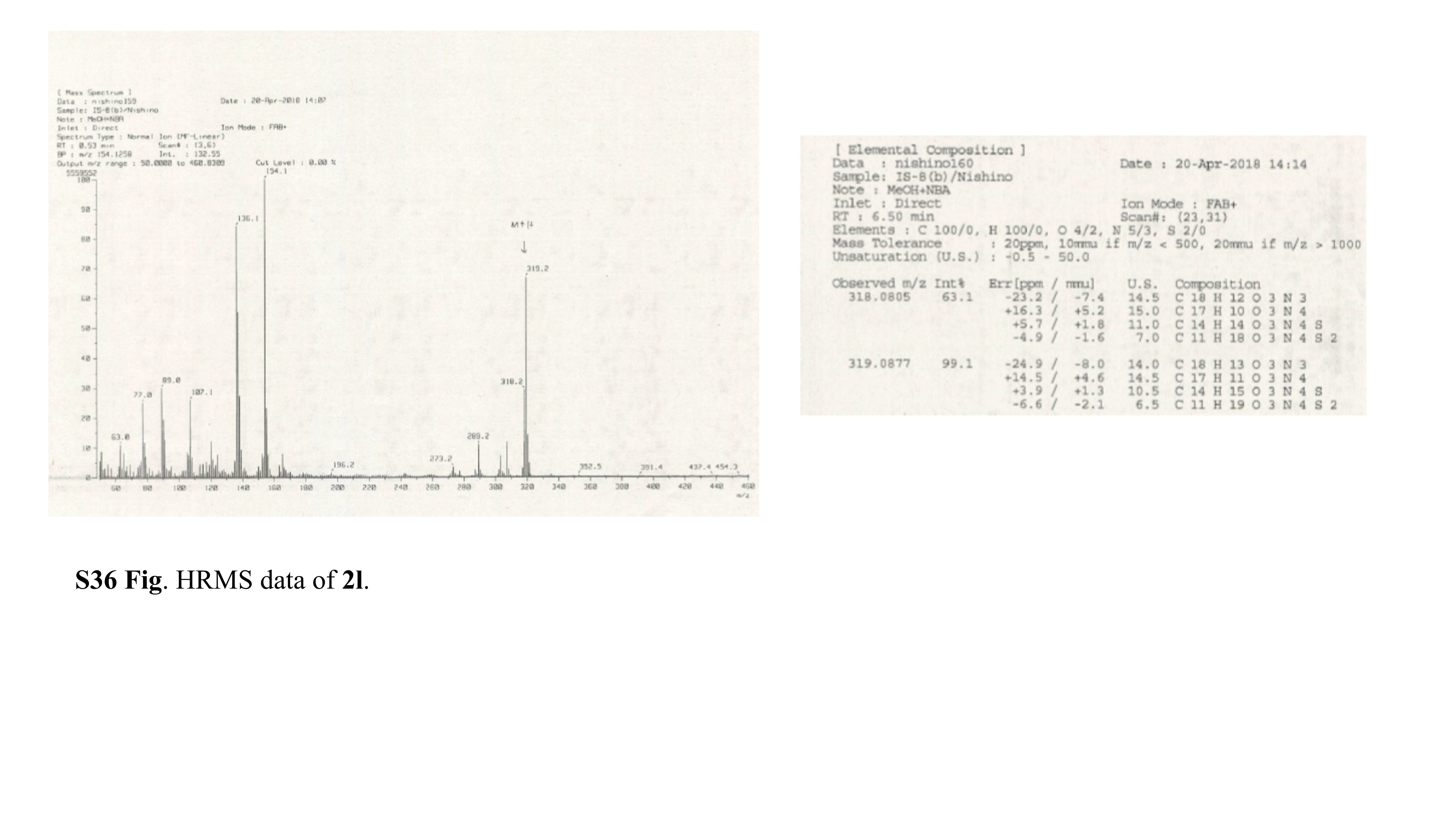

Supplement: S36 Fig — (TIF) [file pone.0318999.s036.tif]

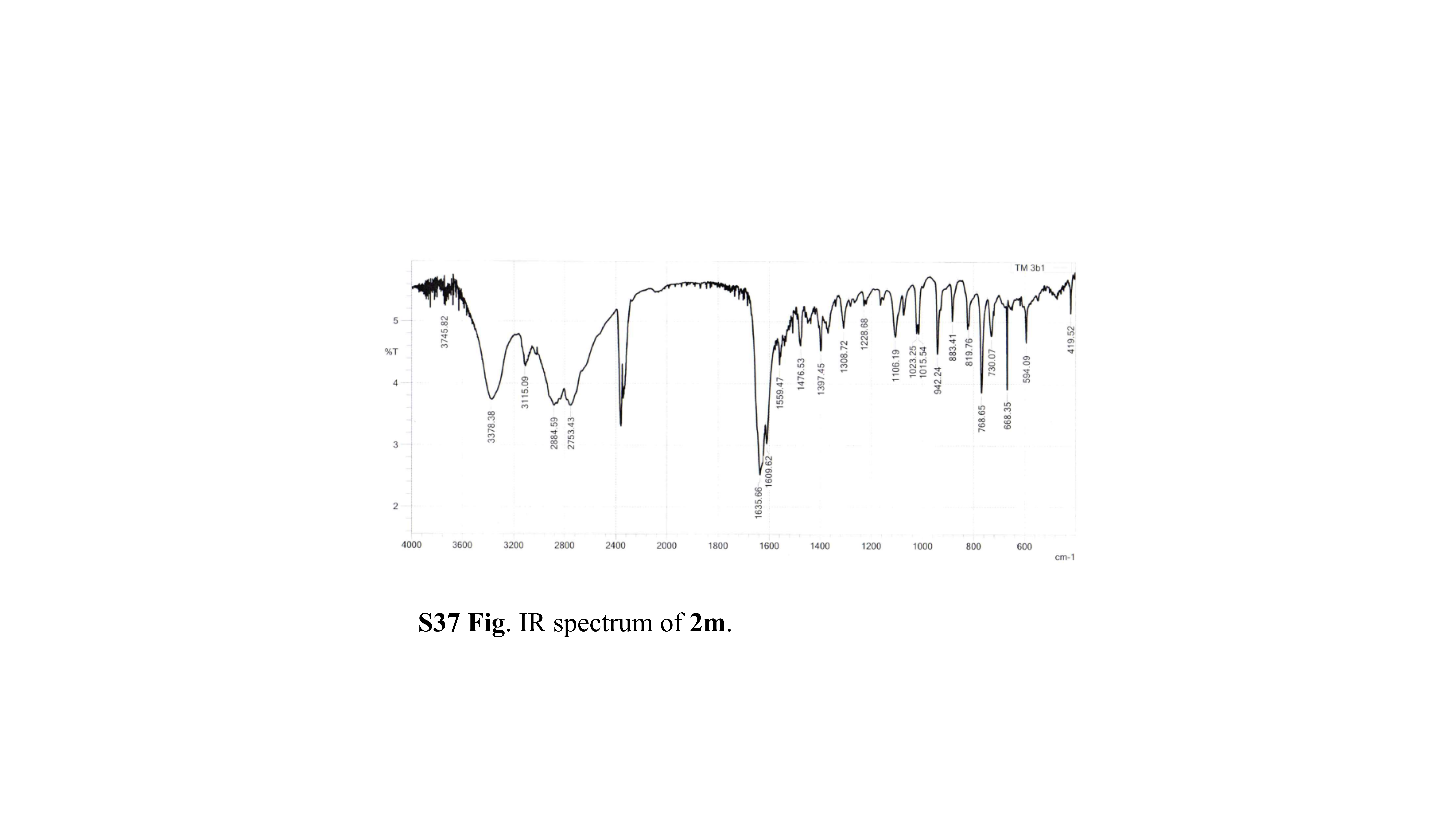

Supplement: S37 Fig — (TIF) [file pone.0318999.s037.tif]

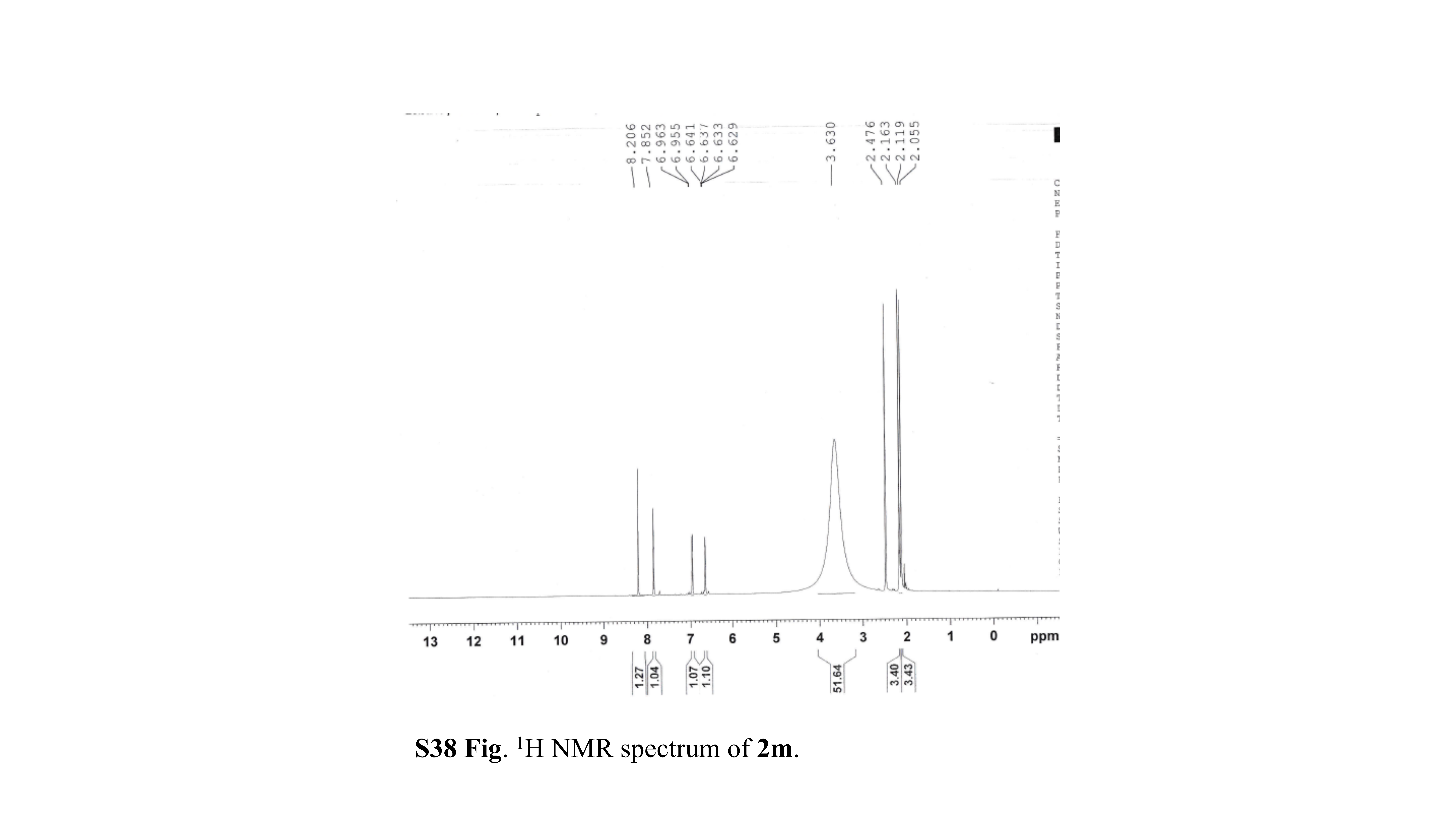

Supplement: S38 Fig — 1H NMR spectrum of 2m. (TIF) [file pone.0318999.s038.tif]

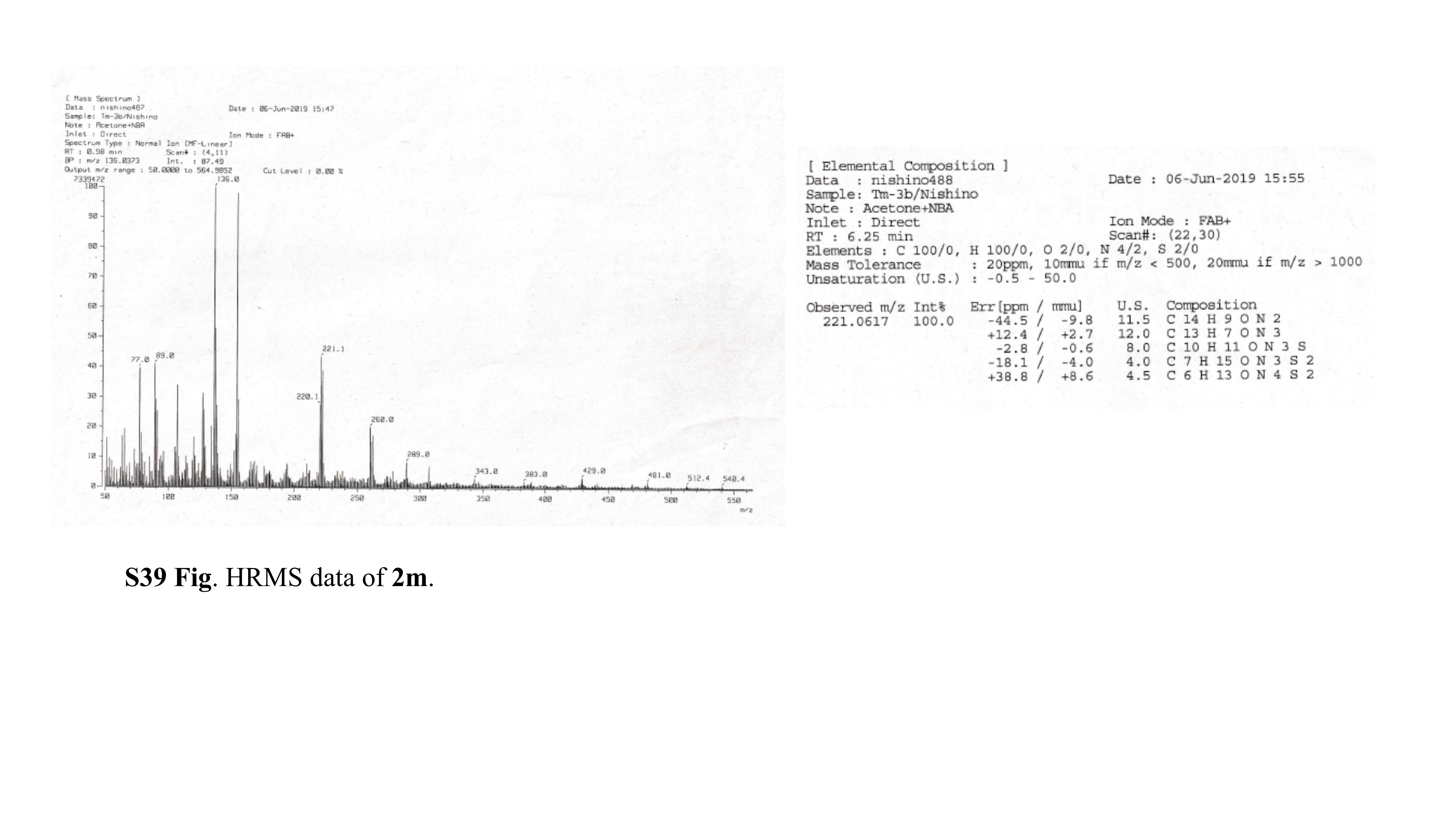

Supplement: S39 Fig — (TIF) [file pone.0318999.s039.tif]

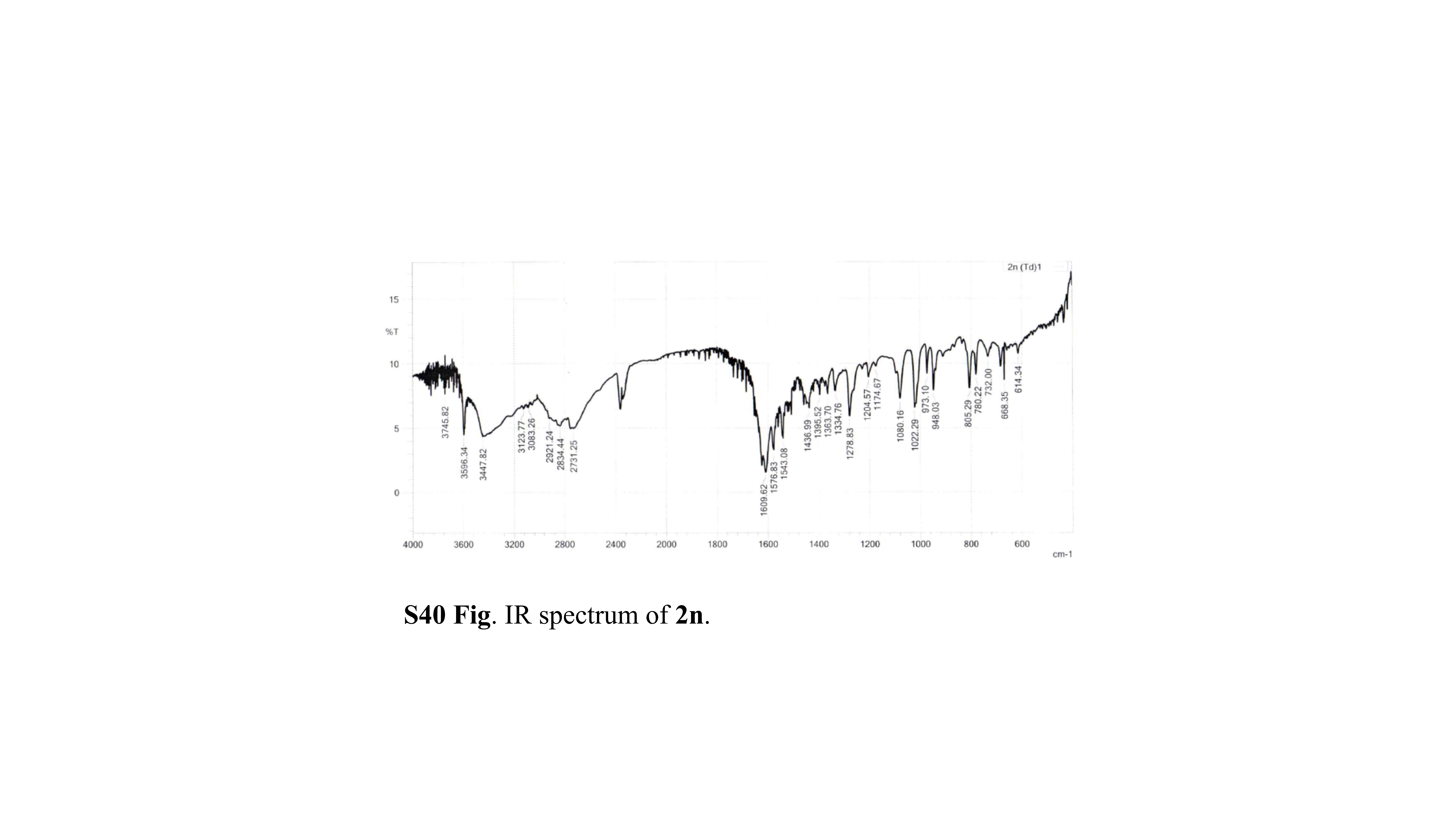

Supplement: S40 Fig — (TIF) [file pone.0318999.s040.tif]

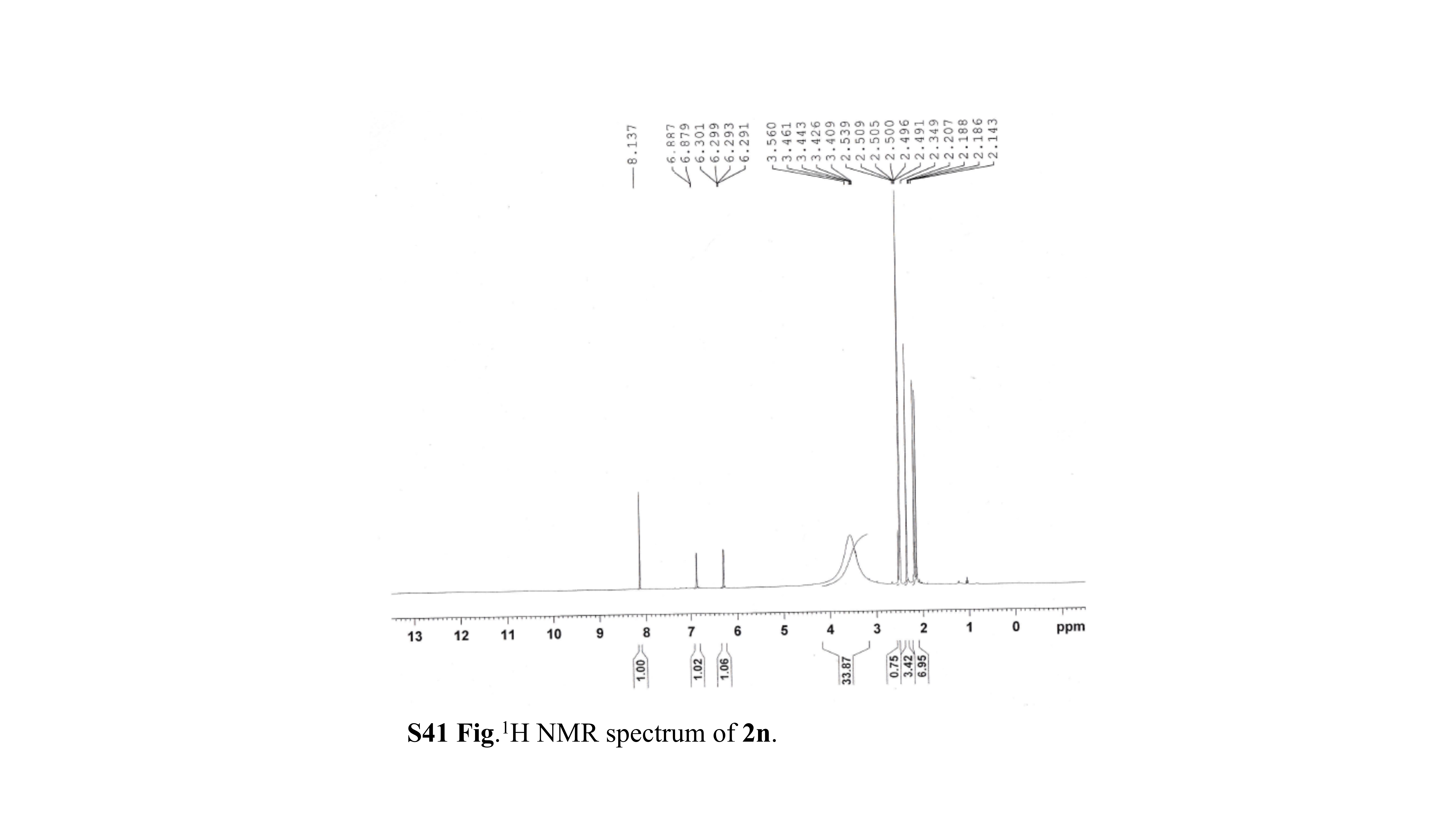

Supplement: S41 Fig — 1H NMR spectrum of 2n. (TIF) [file pone.0318999.s041.tif]

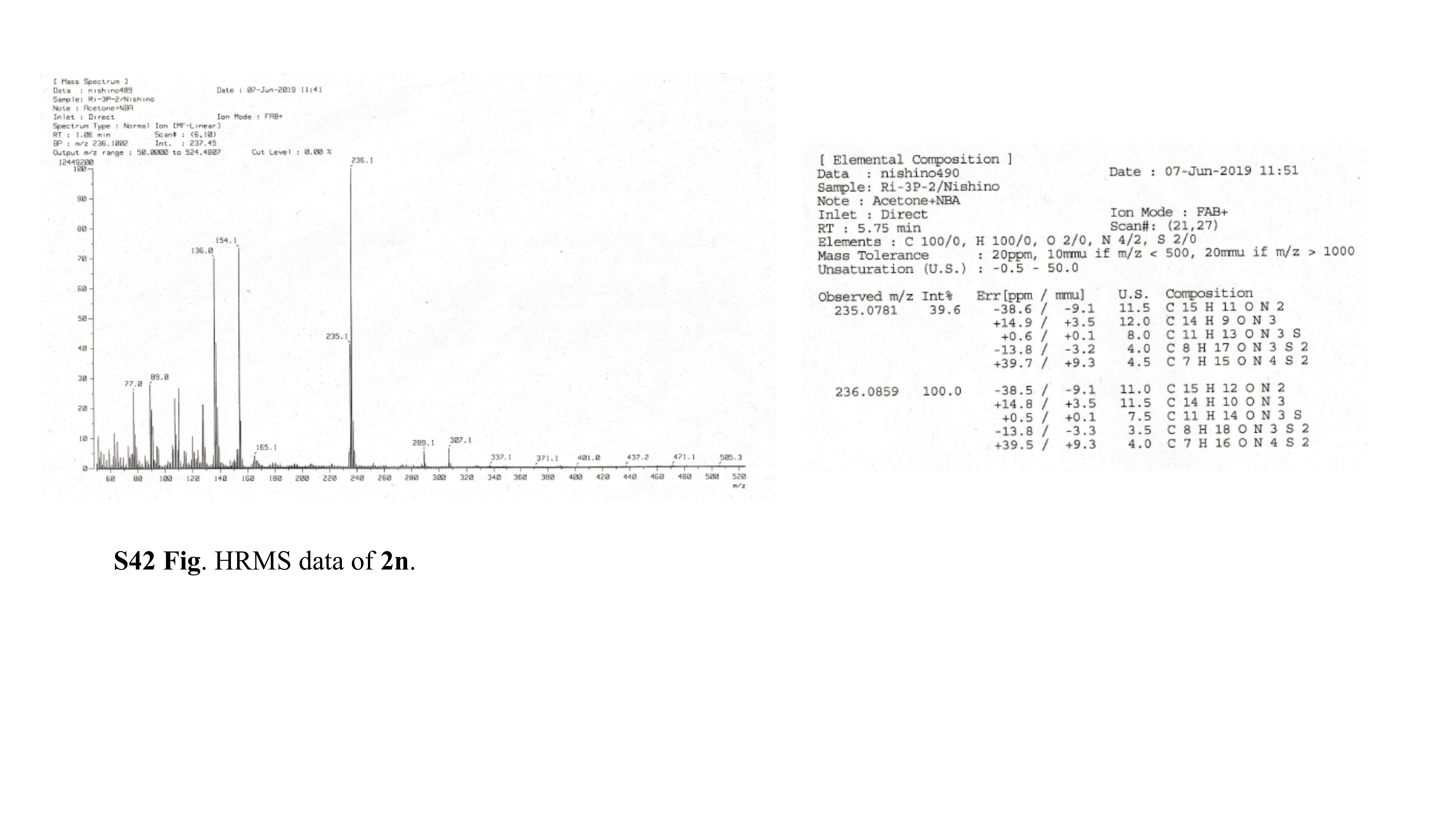

Supplement: S42 Fig — (TIF) [file pone.0318999.s042.tif]

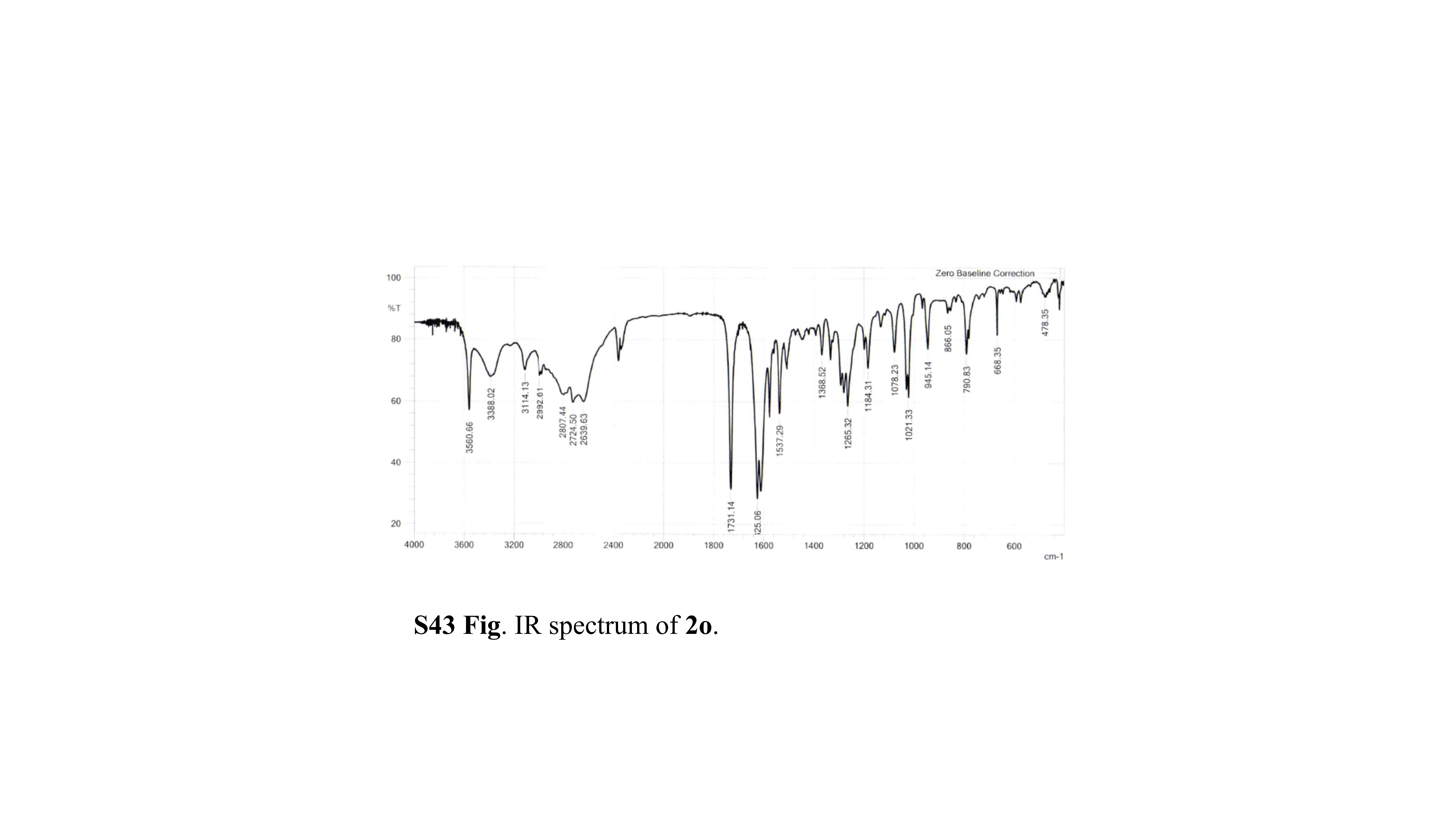

Supplement: S43 Fig — (TIF) [file pone.0318999.s043.tif]

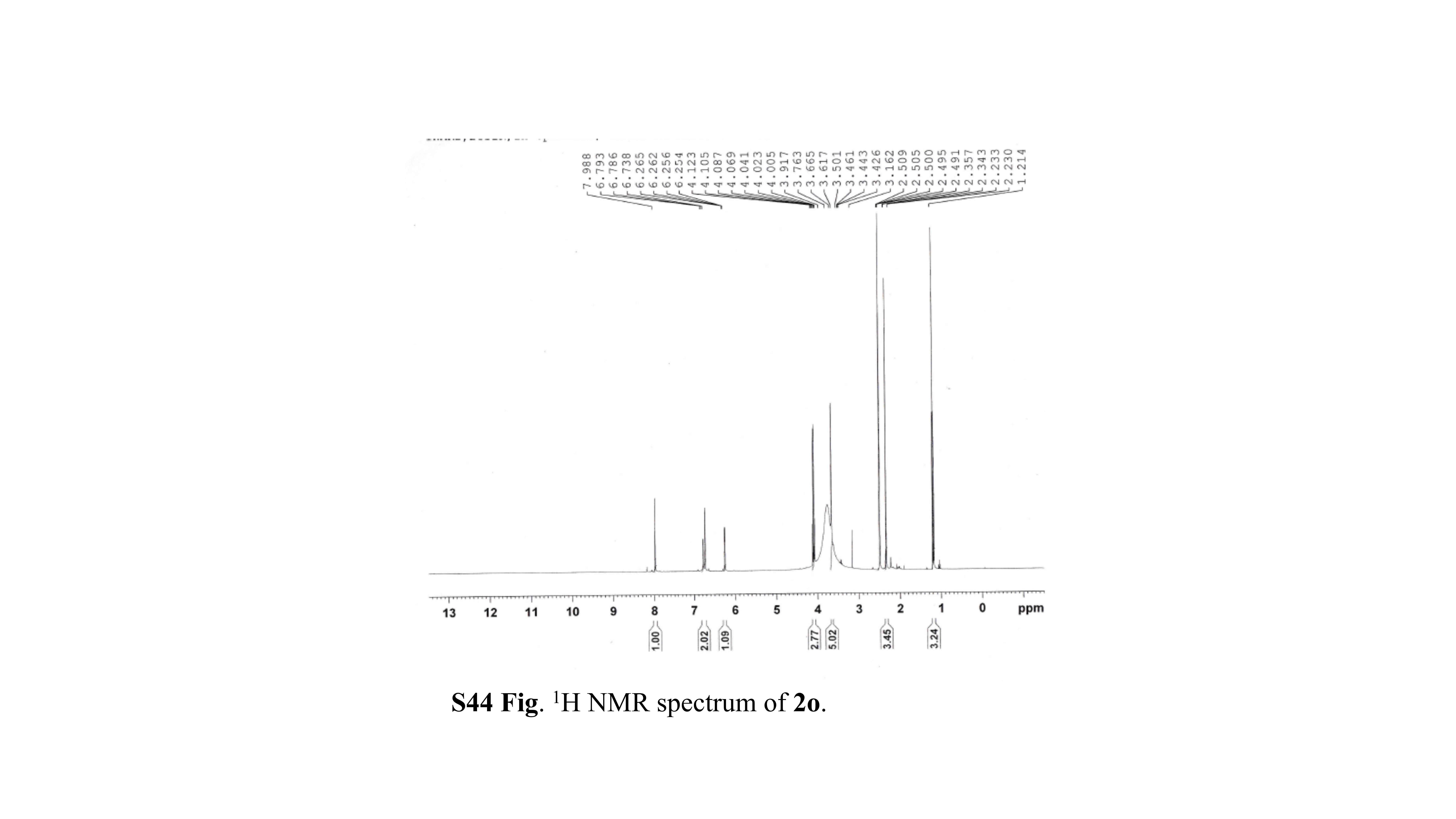

Supplement: S44 Fig — 1H NMR spectrum of 2o. (TIF) [file pone.0318999.s044.tif]

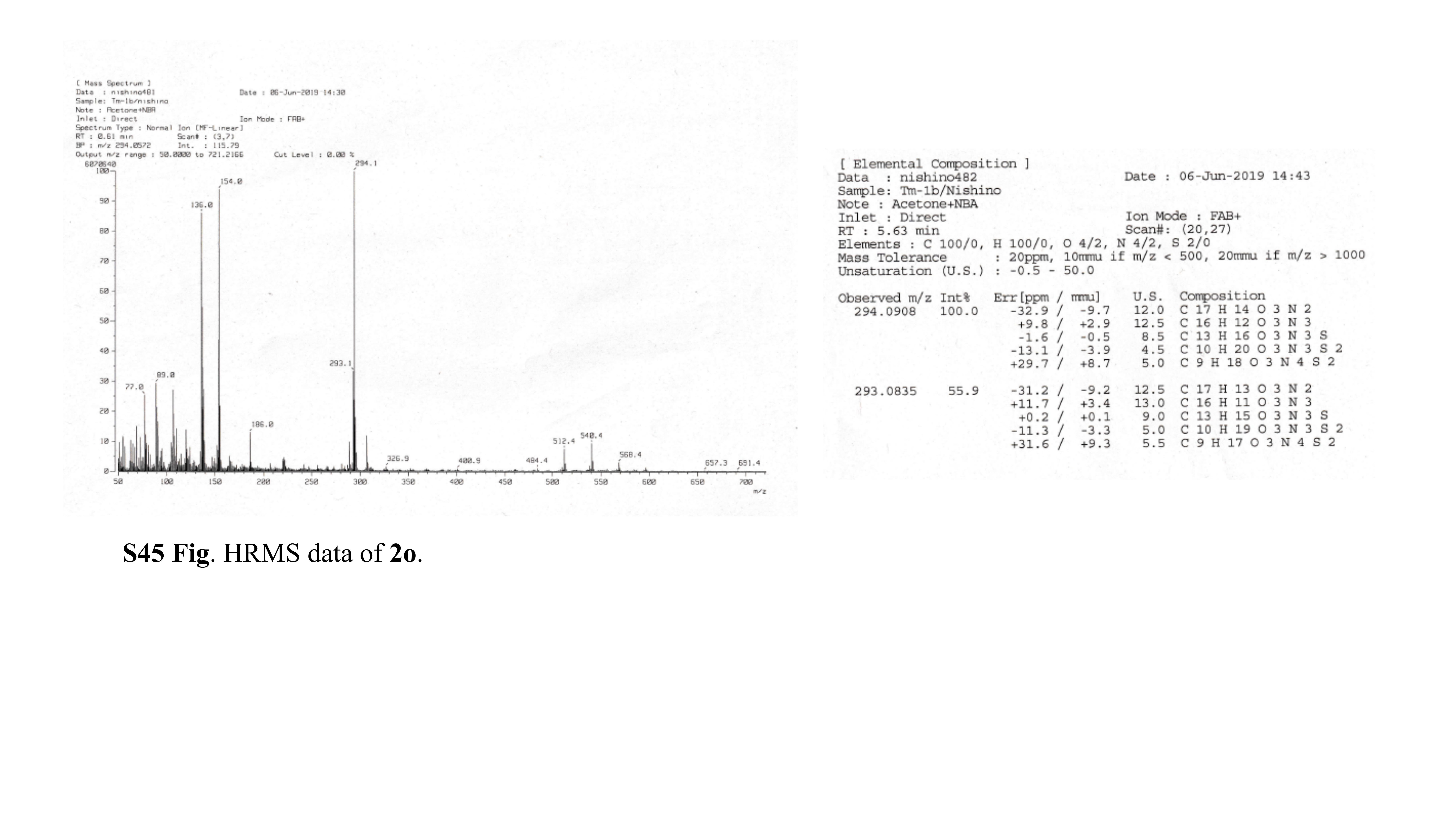

Supplement: S45 Fig — (TIF) [file pone.0318999.s045.tif]

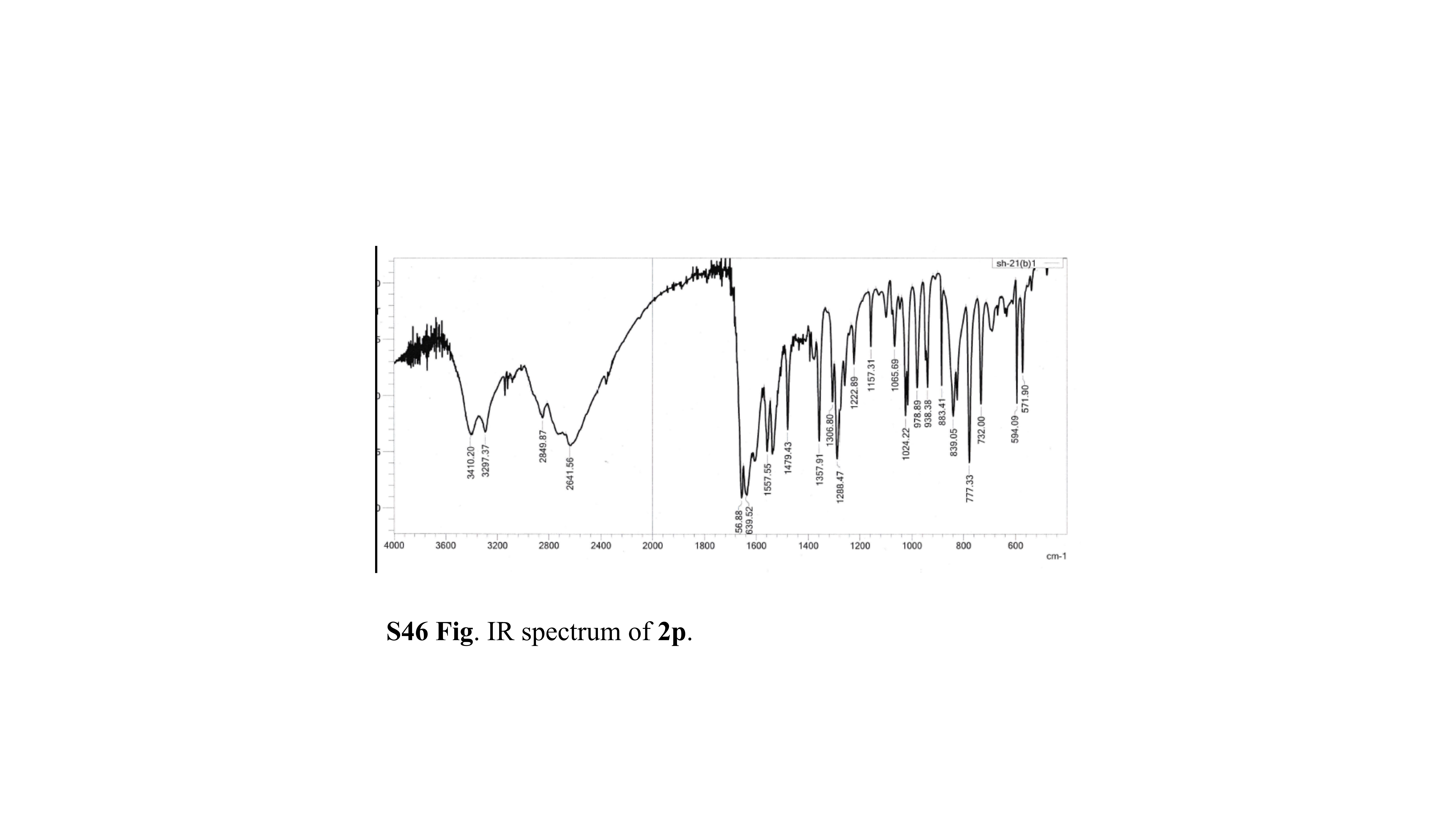

Supplement: S46 Fig — (TIF) [file pone.0318999.s046.tif]

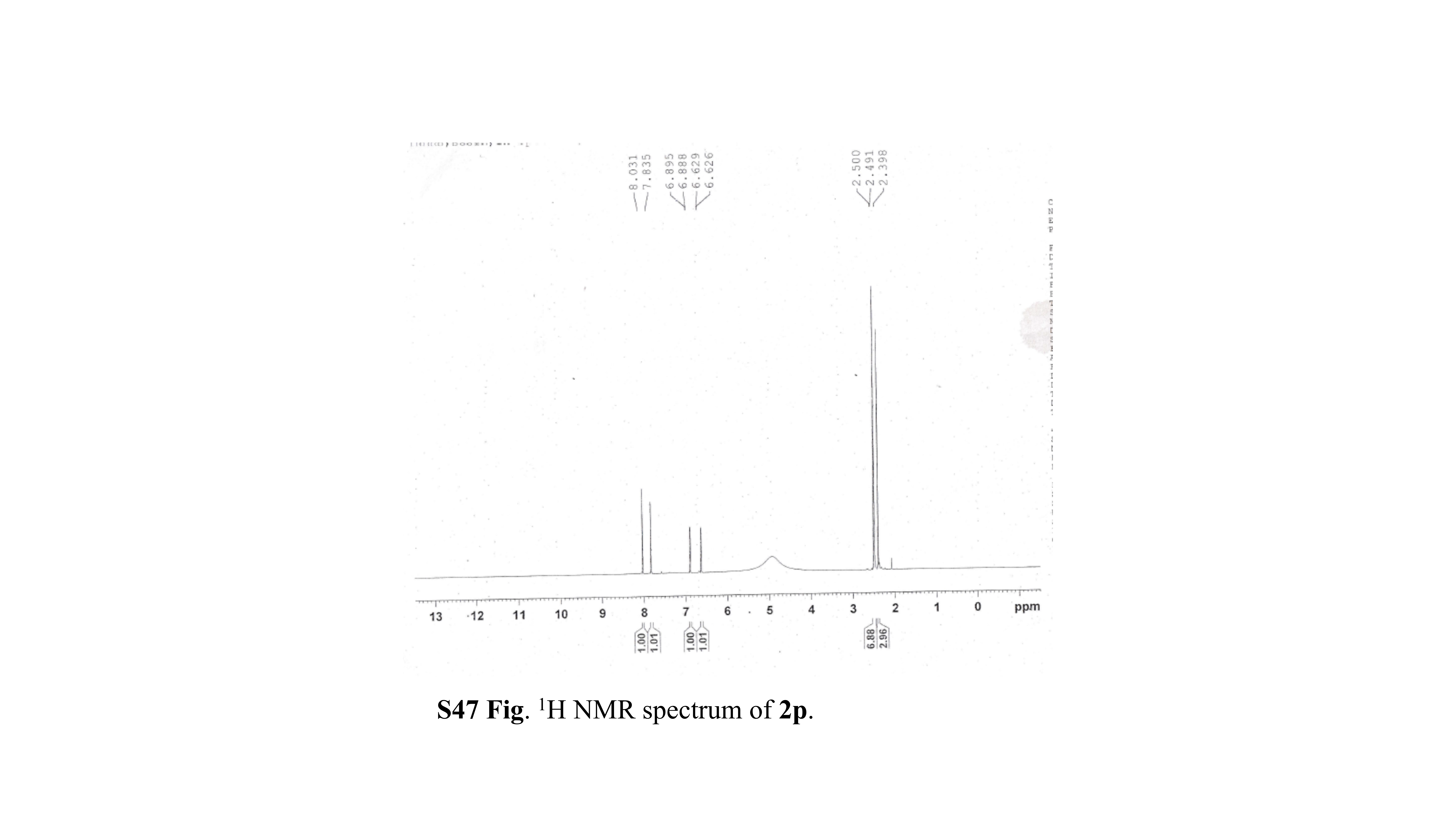

Supplement: S47 Fig — 1H NMR spectrum of 2p. (TIF) [file pone.0318999.s047.tif]

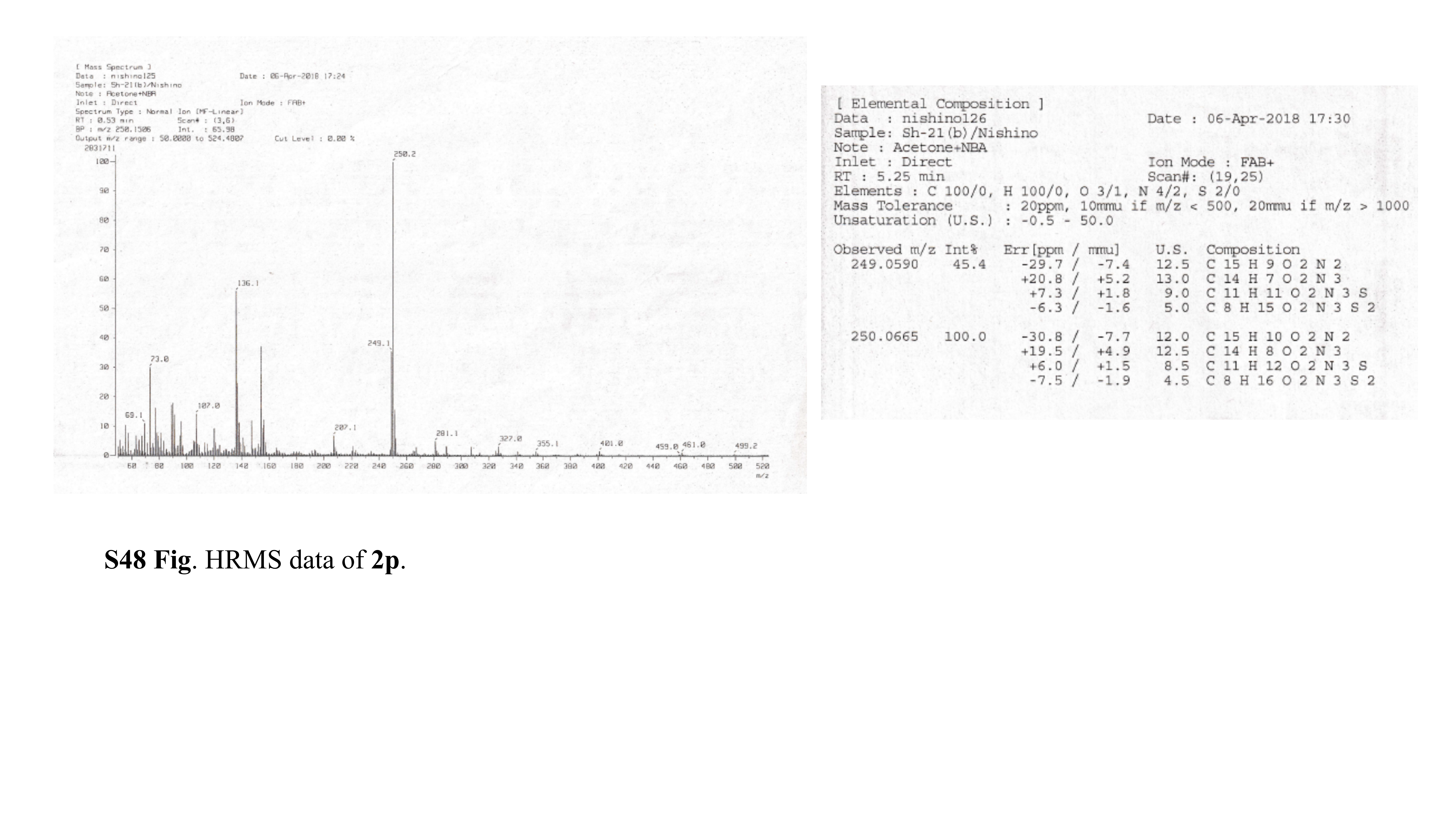

Supplement: S48 Fig — (TIF) [file pone.0318999.s048.tif]

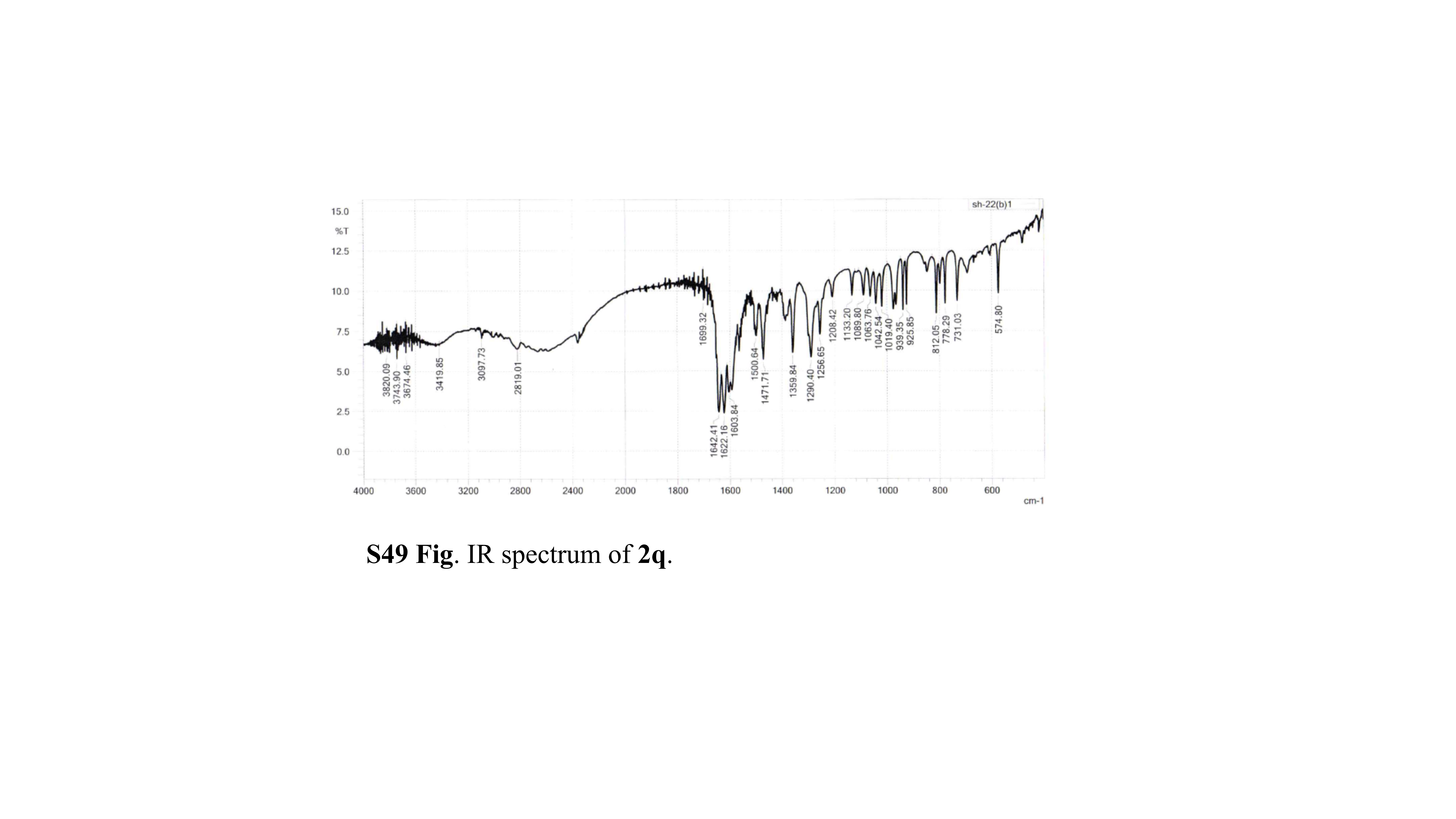

Supplement: S49 Fig — (TIF) [file pone.0318999.s049.tif]

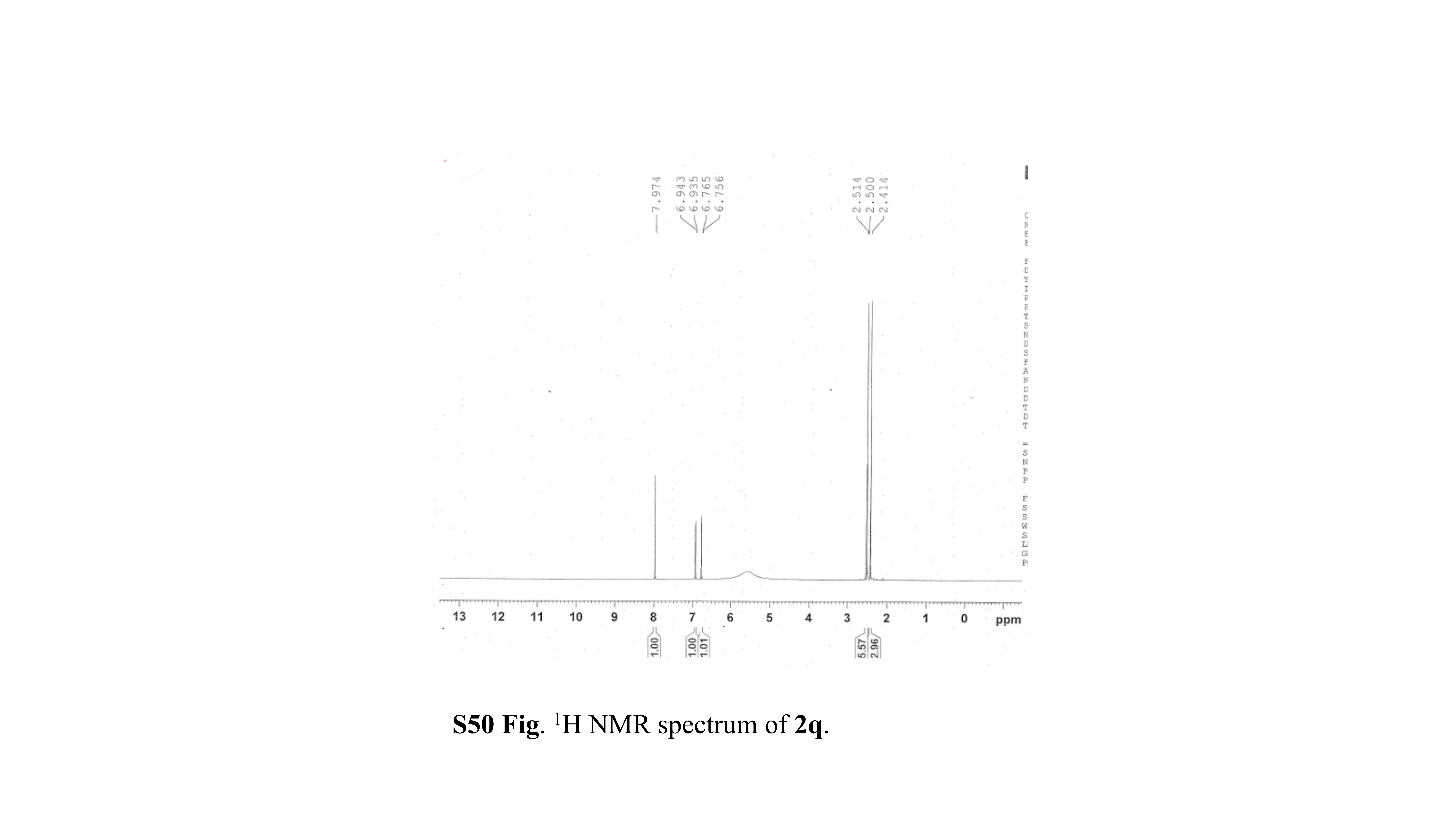

Supplement: S50 Fig — 1H NMR spectrum of 2q. (TIF) [file pone.0318999.s050.tif]

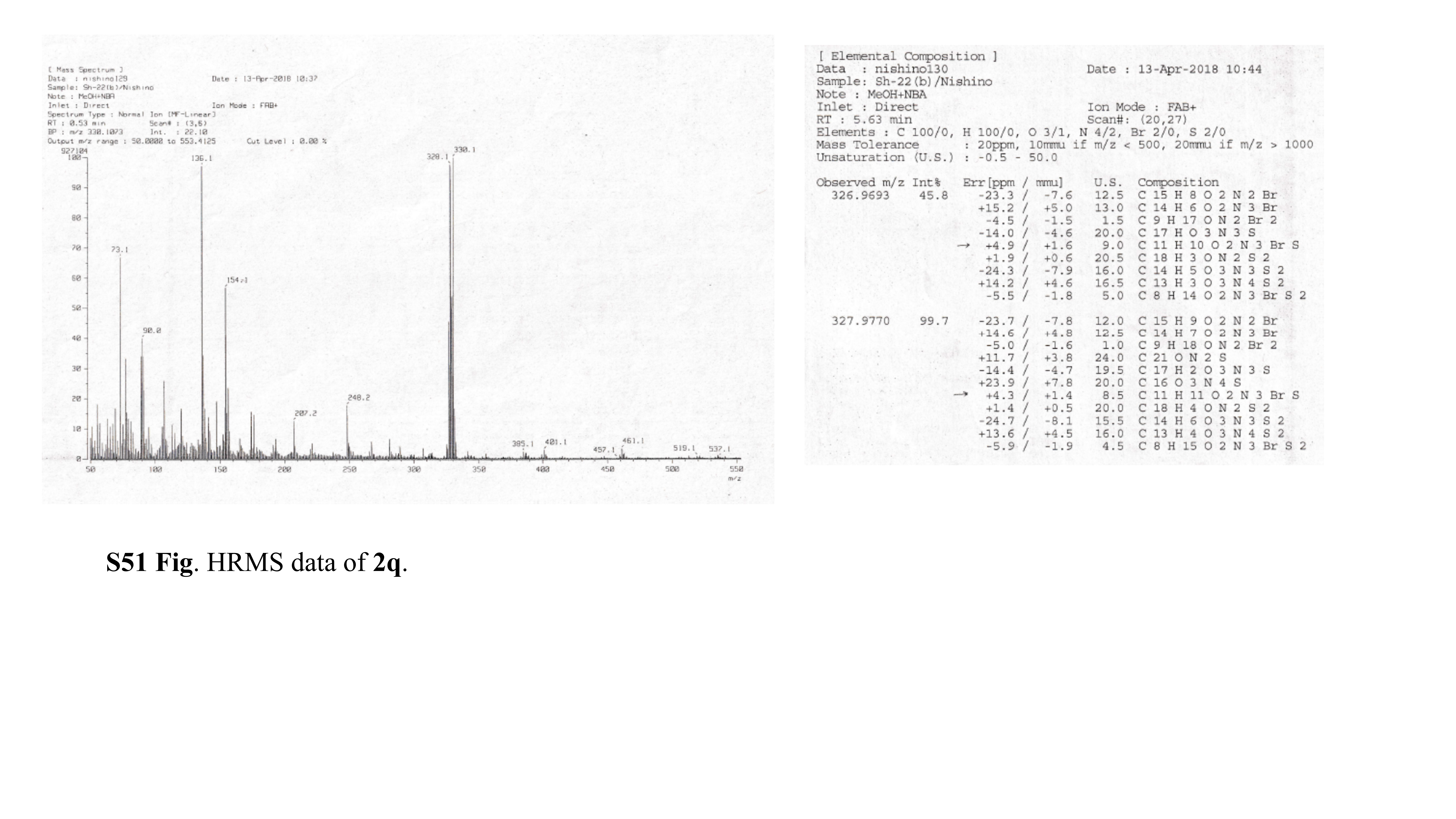

Supplement: S51 Fig — (TIF) [file pone.0318999.s051.tif]

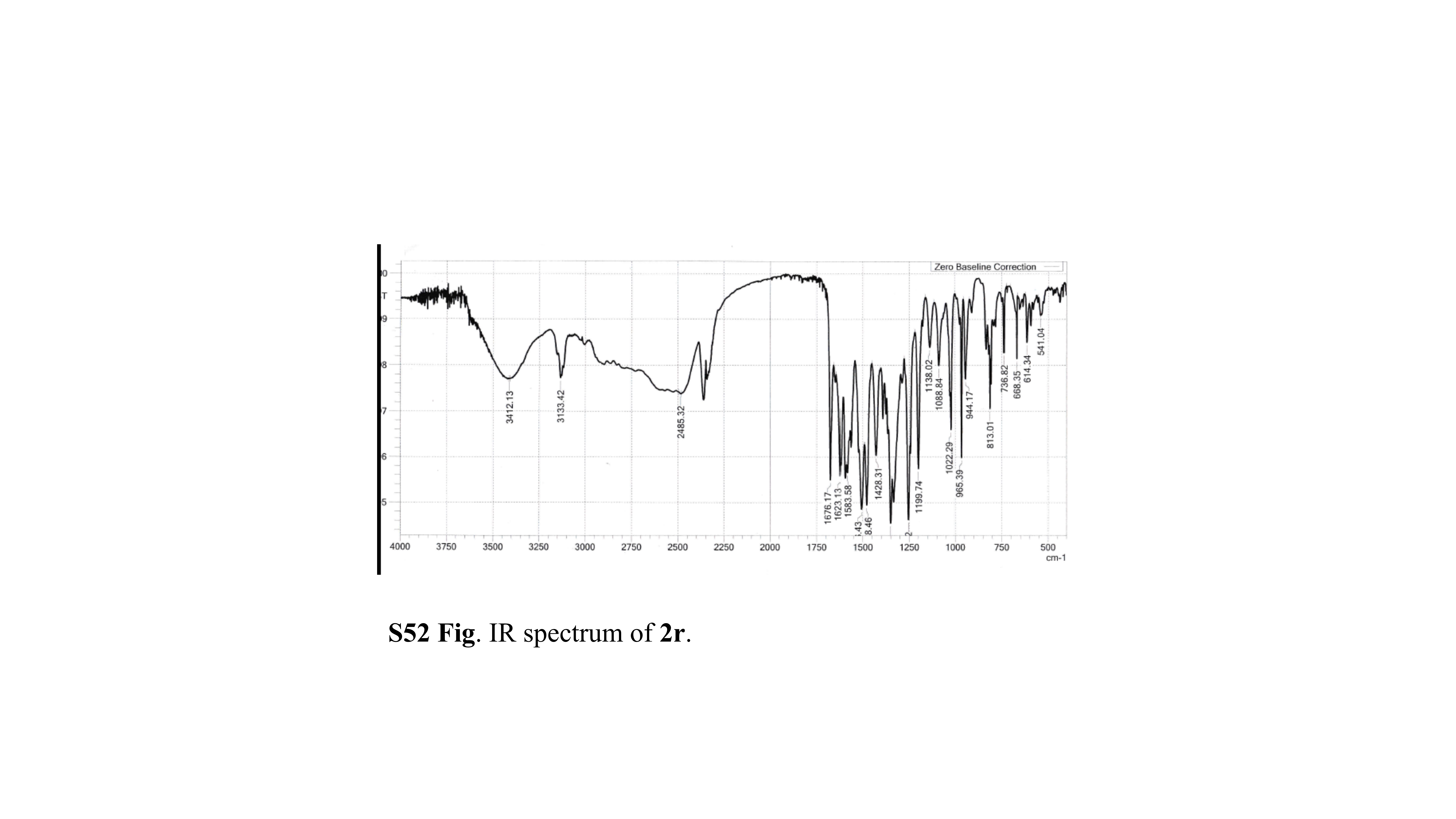

Supplement: S52 Fig — (TIF) [file pone.0318999.s052.tif]

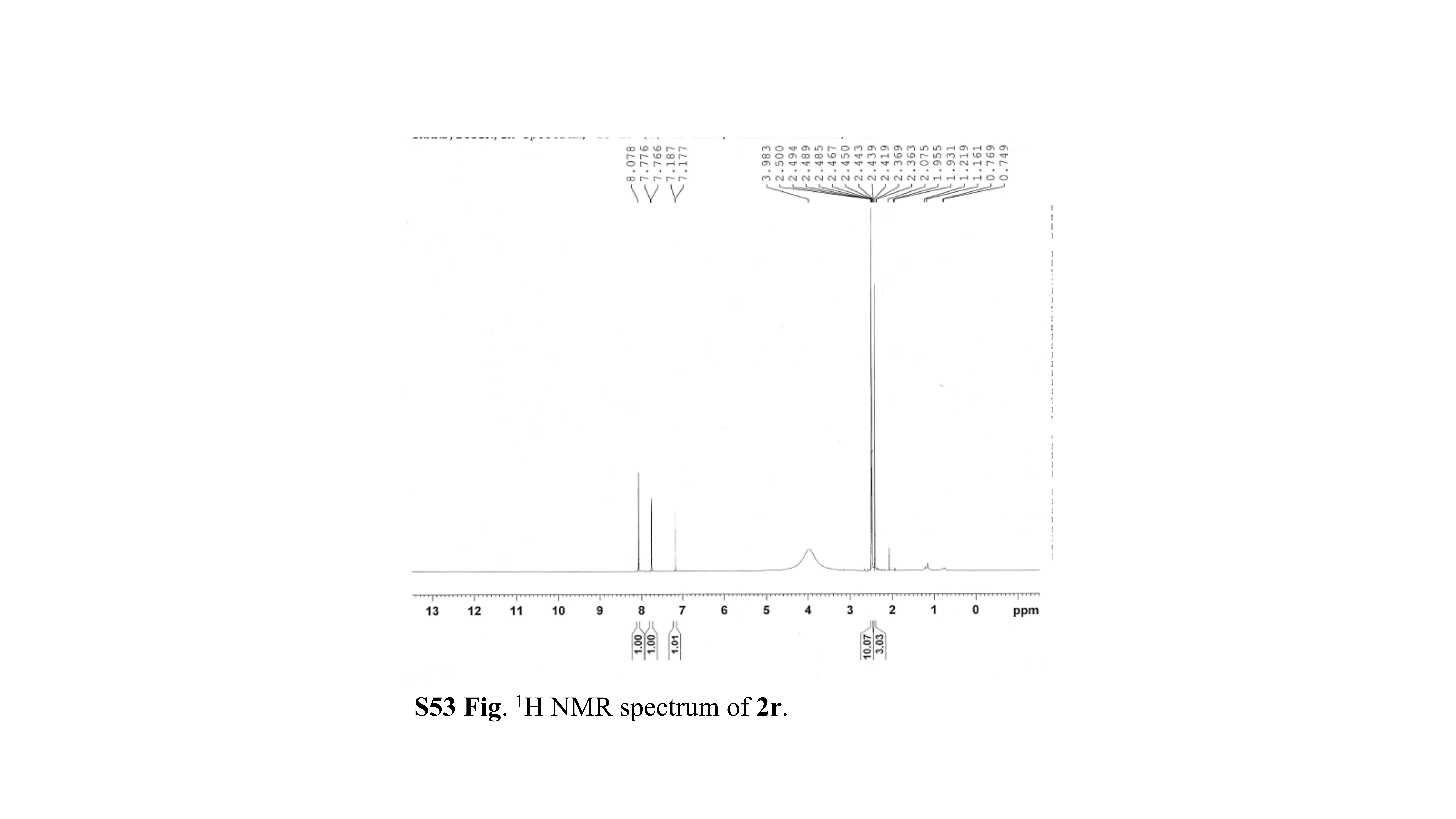

Supplement: S53 Fig — 1H NMR spectrum of 2r. (TIF) [file pone.0318999.s053.tif]

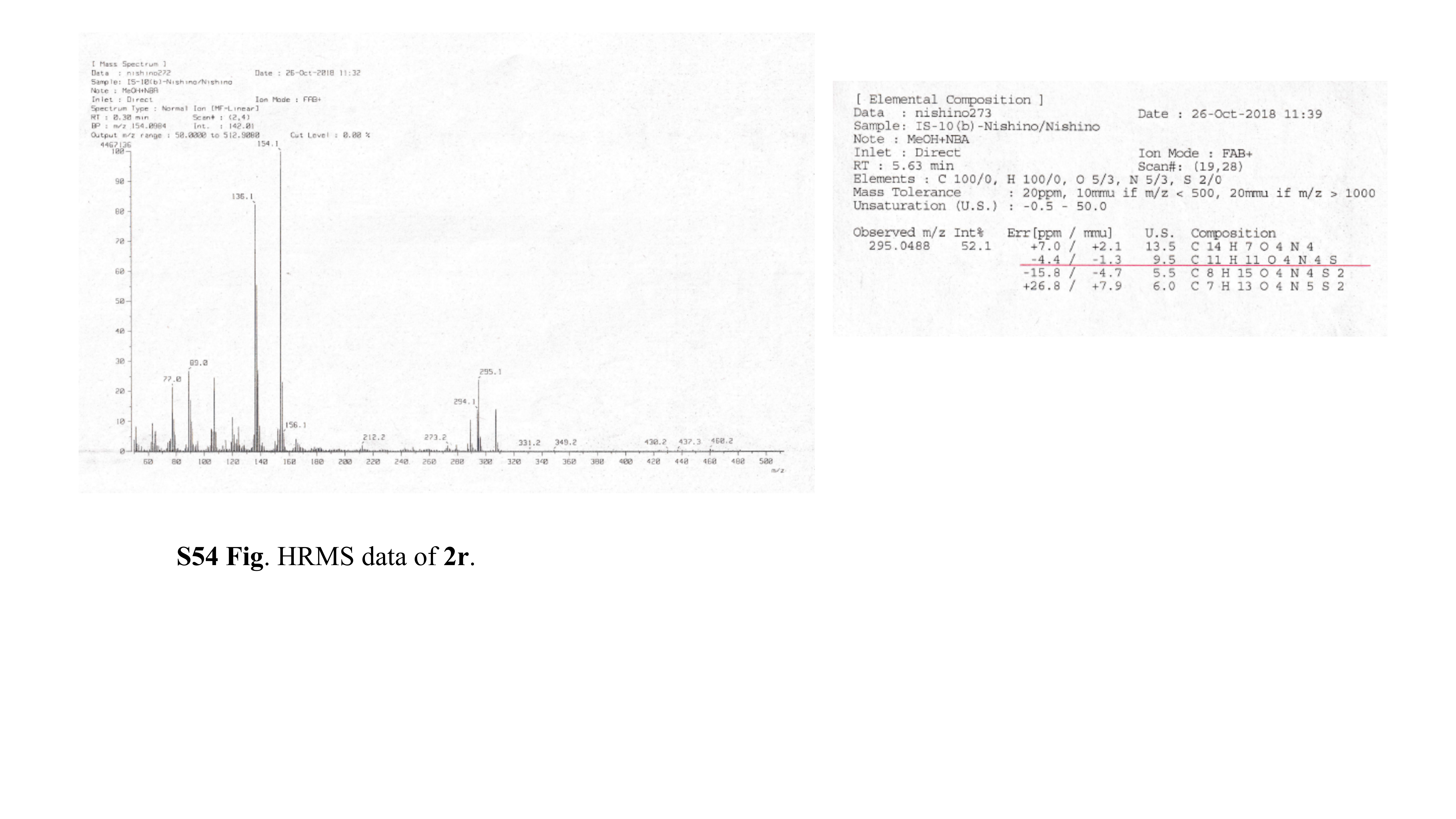

Supplement: S54 Fig — (TIF) [file pone.0318999.s054.tif]
