# Supplementary material for: Synthesis, antibacterial activity, in silico ADMET prediction, docking, and molecular dynamics studies of substituted phenyl and furan ring containing thiazole Schiff base derivatives
Source: PLoS One. 2025 Mar 10;20(3):e0318999. doi: 10.1371/journal.pone.0318999 (PMC11892886; doi:10.1371/journal.pone.0318999)
Supplement: Attachment — Submitted filename: Comments letter.docx [file pone.0318999.s055.docx]

**Comments**

1) In the abstract, there are terms like ‘conspicuously’. It would be better to use some scientific terms instead of these terms.
2) It would be better to use the compound numbers as **2(a-l)** instead of **2a-2l**. Wherever applicable, it should be corrected.
3) English and grammar corrections should be done with utmost care. Overall manuscript should be revised in these aspects.
4) References section also should be rechecked according to the journal guidelines.

5)	Plagiarism should be checked.

6)	Schiff base synthetic approaches are really commendable and results are promising.

7)	Manuscript has many merits.

**Report**

After addressing the above comments, I would strongly recommend to “accept and publish” in “PlosOne’.
