# Supplementary material for: Synthesis, antibacterial activity, in silico ADMET prediction, docking, and molecular dynamics studies of substituted phenyl and furan ring containing thiazole Schiff base derivatives
Source: PLoS One. 2025 Mar 10;20(3):e0318999. doi: 10.1371/journal.pone.0318999 (PMC11892886; doi:10.1371/journal.pone.0318999)
Supplement: Attachment — Submitted filename: Response to Reviewers_PONE-D-24-40454.docx [file pone.0318999.s056.docx]

**Response to Reviewers (PONE-D-24-40454)**

**Response to Reviewer #1**

1. The introduction lacks a detailed discussion on the novelty of the synthesized thiazole Schiff base derivatives. More emphasis is needed on how these compounds stand out from other similar derivatives in terms of structure and biological activity.

**Response:** Several sentences with references were added in introduction section regarding the novelty of the synthesized compounds and highlighted with yellow marks.

2. The rationale behind selecting the specific aryl and heteroaryl derivatives for synthesis is not clearly explained. A brief justification of the choice of functional groups and their expected effects on biological activity would strengthen the manuscript.

**Response:** Several sentences with references were added in the introduction section regarding the rationale behind selecting the specific aryl and heteroaryl derivatives for synthesizing the new derivatives and highlighted with yellow marks.

3. The discussion on how the thiazole Schiff base derivatives interact with the bacterial cell wall or proteins is not sufficiently detailed.

**Response:** Several sentences were written in the results and discussion section (Molecular docking studies) with reference to clarify the interaction of the derivatives with the bacterial cell wall and marked in yellow.

4. Some synthesized compounds, such as 2e, 2g, and 2h, showed no activity, but there is no thorough discussion of why this might be the case. A comparison between active and inactive compounds in terms of structural differences could help explain these results.

**Response:** According to the reviewer's comments, the antimicrobial activity part in results and discussion section was re-written with references to explain the structure-activity relationship and highlighted with yellow color in the revised manuscript.

5. The molecular docking section lacks clarity on how the protein receptors (6JHK for B. subtilis and 1KZN for E. coli) were selected. Explaining why these receptors are relevant targets for the synthesized compounds would make the docking studies more compelling.

**Response:** Several sentences regarding the docking validation of the ligands against the respective receptors were added and highlighted with yellow color (in molecular docking sub-section of results and discussion section).

6. While the manuscript includes DFT calculations for structure optimization, there is no detailed explanation of the relevance of these calculations to the overall study. Clarifying how DFT helps in understanding the molecular properties of the synthesized compounds would be beneficial.

**Response:** According to the reviewer’s comment, several sentences were added in the revised manuscript to discuss the relevance of DFT method with references in the Experimental section, which is marked in yellow.

7. The ADMET prediction results are presented in tabular form, but the implications of these results for the potential development of oral drugs are not adequately discussed. More analysis is needed to highlight the importance of ADMET properties like %ABS and toxicity.

**Response:** The ADMET prediction results were explained adequately to make the tabular data understandable. Moreover, a sentence was added in the results and discussion section (In silico ADMET prediction) to make it clearer and highlighted with yellow mark.

8. There is no mention of statistical tests used to analyze the antibacterial data. Applying statistical analysis would add rigor to the comparisons between different compounds and standard antibiotics.

**Response:** We used “agar disk diffusion” method to analyze the antibacterial potency of the synthesized molecules and compared them against the standard ceftriaxone, which is represented in Table 1 and discussed in the result and discussion section. All experiments were repeated three times and data were taken as mean ± standard deviation which was mentioned as footnote under the table.

9. Why only this particular basis set has been used for DFT? Improvement is required in the theoretical section. Go through the following recent articles with DFT/docking/MD and support the manuscript with the following references to make it more- 10.1016/j.ijleo.2022.169367; 10.1080/07391102.2022.2162583; 10.1016/j.molliq.2024.124412; <https://dx.doi.org/10.4314/bcse.v37i2.19>

**Response:** Several sentences were added in the revised manuscript to discuss the relevance of the DFT method with the above-mentioned references in the experimental section and marked in yellow.

**10.** The conclusion is concise but lacks a forward-looking perspective. Expanding on potential future directions, such as exploring the clinical relevance of the synthesized compounds or testing them in more complex biological systems, would strengthen the conclusion.

**Response:** Several sentences regarding the forward-looking perspective were added in the conclusion section and marked in yellow.

**Response to Reviewer #2**

Comments
1) In the abstract, there are terms like ‘conspicuously’. It would be better to use some scientific terms instead of these terms.

**Response:** The word ‘conspicuously’ was replaced by the word ‘prominently’ according to the reviewer's comments and marked in yellow.

2) It would be better to use the compound numbers as 2(a-l) instead of 2a-2l. Wherever applicable, it should be corrected.

**Response:** Compound numbers were re-written in the revised manuscript according to the reviewer's comments and marked them in yellow.

3) English and grammar corrections should be done with utmost care. Overall manuscript should be revised in these aspects.

**Response:** We have gone through the manuscript and corrected the grammatical and typographical errors in the revised manuscript, where found and highlighted with yellow color.

4) References section also should be rechecked according to the journal guidelines.

**Response:** We re-checked the references attentively, organized them according to the Journal’s rules, and marked them with yellow.

5) Plagiarism should be checked.

**Response:** After revise the manuscript, plagiarism was checked by **iThenticate** software. No significant plagiarism was detected. Mostly spectral data, figure captions, affiliations, title of different sections and sub-sections, references, and some scientific common terms were detected during checking.

**Response to Reviewer #3**

1- I suggest to modify the title.

**Response:** The title of the manuscript was modified according to the reviewer’s comment to make it more specific. The term ‘aryl/heteroaryl’ was replaced by ‘substituted phenyl and furan’ in the revised title and marked in yellow.

2- Codes of the synthesized compounds must be in the bracket

**Response:** Codes of the compounds were re-written in the revised manuscript according to the reviewer's comments and marked in yellow.

3- It is suggested to reduce and duplications, such as Scheme 2 and Scheme 3, can write as Schemes 2 and 3.

**Response:** It was corrected accordingly and marked them in yellow in the revised manuscript.

4- Abstract section is weak. Please improve it and add problem statement/research gap.

**Response:** Abstract section was re-written mentioning research gap in the revised manuscript and marked in yellow.

5- The authors should investigate MIC studies for the compounds. Only inhibition zone determinations is not enough for recent studies.

**Response:** According to reviewer’s comment, the minimal inhibitory concentrations (MICs) of the two most active compounds **2d** and **2n** were determined against *B. subtilis* bacterial strain. The values were added in the results and discussion section (Antibacterial activity) and highlighted in yellow.

6- How active site of the target receptors are determined? The have co-crystalized ligand, authors should use to validated the reliability of docking procedure.

**Response:** We have considered the receptor protein’s calculated structure where the ligand interacted and the obtained structure’s binding site to determine the active sites and validate the docking with the respective proteins.

7- Some references for biological activities, and computational parts are recommended: 10.2174/1877946812666220928102954, <https://doi.org/10.1021/acsomega.4c03679>, <https://doi.org/10.1007/s11164-024-05354-x>, <https://doi.org/10.1021/acsomega.3c08407>

**Response:** Three of the above-mentioned papers were cited in the results and discussion section (Ref. 50–52) as one was not found, and the other reference numbers were changed accordingly.

8- The conclusion should contain more of the quantitative data.

**Response:** According to the reviewer's comment, few sentences regarding the quantitative outcomes of the present work were included in the conclusion section and highlighted in yellow.

9- The whole manuscript needs to be checked to avoid typo-error.

**Response:** We have gone through the manuscript, and linguistic errors were corrected in the revised manuscript where applicable.
